# 3D-Fiber Mapping in Biological Tissues with Computational Scattered Light Imaging Enabled Through 3D-Nanoprinting

**DOI:** 10.3390/bioengineering13101154

**Published:** 2026-10-02

**Authors:** Mark M. Vermeulen, Pieter F. J. van Altena, Dennis Scheidt, Angelo Accardo, Miriam Menzel

**Affiliations:** 1 Department of Imaging Physics, Faculty of Applied Sciences, Delft University of Technology, 2628 CJ Delft, The Netherlands; 2 Department of Precision and Microsystems Engineering (PME), Faculty of Mechanical Engineering, Delft University of Technology, 2628 CD Delft, The Netherlands; p.f.j.vanaltena@tudelft.nl (P.F.J.v.A.); a.accardo@tudelft.nl (A.A.); 3 Institute of Neuroscience and Medicine (INM-1), Forschungszentrum Jülich, 52425 Jülich, Germany; d.scheidt@fz-juelich.de

**Keywords:** light scattering, conical diffraction, optical microscopy, 3D-nanoprinting, two-photon polymerization, biological fibers, fiber mapping, connectivity, nerve fiber network, neuroimaging

## Abstract

Computational Scattered Light Imaging (ComSLI) exploits the anisotropic scattering on aligned structures to reconstruct biological fiber networks like nerve, muscle or collagen fibers. ComSLI discerns individual fiber pathways also in densely interwoven regions with multiple crossing fibers, achieves micrometer resolution across centimeter fields of view, is compatible with various sample preparations and staining, and only requires an LED light source and a camera, making it attractive for histological tissue analysis. However, while ComSLI precisely determines the 2D (in-plane) fiber orientations, determining the 3D (out-of-plane) fiber inclinations remained challenging due to missing a-priori knowledge of the inclination angles. In this study, we measured differently inclined 3D-nanoprinted fiber bundles with ComSLI, compared the resulting scattering patterns to those obtained from nerve fibers in a tilted brain section (vervet corpus callosum), and analyzed scattering patterns and angular profiles in differently inclined brain regions. We show that the highest scattering intensity occurs along the same curved line as conical diffraction at an inclined grating. With this analytical model, we were able to estimate inclination angles of non-crossing fibers up to 60° inclination with an accuracy of 3° or better. This finally allows to quantitatively determine fiber inclinations and reconstruct 3D fiber pathways with ComSLI.

## 1. Introduction

The human body contains many different types of fibrous structures, such as muscle fibers, collagen fibers, or nerve fibers. Skeletal muscle fibers stabilize joints and allow us to move our body, making up approximately 40% of the total body weight [1]. Collagen fibers are a major structural component of connective tissues, including bone, cartilage, tendon, ligament, fascia, and skin [2]. Accurately mapping the orientations of collagen fibers is particularly relevant to cancer research, where collagen organization relative to the tumor boundary has been identified as a prognostic biomarker of disease progression: In breast cancer, the presence of aligned, straightened collagen fibers oriented approximately perpendicular to the tumor boundary has been associated with increased tumor invasion and reduced survival [3]. Nerve fibers (axons with/without myelin sheath) carry electrical signals between the nervous system and other tissues, and can be found throughout the entire body. One of the most complex biological fiber networks can be found in our brain where billions of nerve cells are interconnected by nerve fibers with an estimated total length of approximately 2–3 million kilometers [4]. Mapping the pathways of these densely interwoven fibers with high precision is required for better understanding the brain’s function in health and disease, and remains a current challenge for neuroimaging techniques.

There exist various techniques to analyze fibrous tissues which all come with different limitations: Serial-scanning electron microscopy [5,6,7] visualizes biological structures at nanometer-scale resolution and allows to trace the fiber pathways across adjacent sections through image segmentation, which is computationally demanding and labor-intensive, limiting the volume that can be analyzed. Conventional light microscopy combined with histological staining or fluorescent labeling [8,9,10,11] allows to study larger fields of view, while techniques such as light-sheet fluorescence microscopy [12], two-photon fluorescence microscopy [13], or optical coherence tomography (OCT) [14] even enable the visualization of fibrous tissues in 3D, but they can also only indirectly compute the fiber orientations, e.g., via structure tensor analysis, and they are inaccurate in densely packed fiber regions with insufficient contrast between fibers.

To quantitatively reconstruct fiber pathways/orientations also in densely packed tissues, there exist other techniques that allow to directly map the fiber pathways by exploiting (optical) tissue properties: Diffusion magnetic resonance imaging (dMRI) [15,16,17,18] exploits the anisotropic diffusion of water molecules in fibrous tissues to reconstruct fiber pathways also in regions with multiple crossing fibers. It can be applied to whole brain volumes in vivo, but the resolution is insufficient to resolve individual fibers with micrometer diameter. Polarization-based techniques such as polarization-sensitive OCT (PS-OCT) or 3D-polarized light imaging (3D-PLI) exploit the birefringence properties of biological fibers to compute their orientations. While PS-OCT can be applied to 3D-volumes [19,20], 3D-PLI reconstructs 3D-fiber orientations in 2D-tissue sections with micrometer resolution and square-centimeter fields of view [21,22]. However, both techniques only yield a single fiber orientation per tissue voxel, are limited in correctly determining the orientations of out-of-plane and multiple crossing fibers [23,24,25], and can only be applied to tissues with sufficient birefringence contrast: Polarization microscopy has, for example, difficulties with reconstructing white matter fiber pathways in formalin-fixed paraffin-embedded (FFPE) tissues [26,27] which are commonly used in histo(patho)logy, and it has been shown to yield slightly different collagen/muscle fiber orientations for differently stained FFPE sections [27]. Furthermore, it is not always compatible with common immunohistochemistry fluorescence stains, e.g., Triton X-100 has been shown to impair the birefringence of myelinated nerve fibers [28]. Polarimetric second harmonic generation (pSHG) accurately determines muscle or collagen fiber orientations, also in regions with multiple crossing fibers and in stained FFPE sections [29], but it is not suited for directly visualizing nerve fibers and is restricted to smaller fields of view. Small-angle X-ray scattering (SAXS) exploits the anisotropic scattering produced by aligned (nano)structures, e.g., the layered structure of the nerve myelin sheath [30] or ordered collagen fibrils [31], to reconstruct the fiber orientations, independent of birefringence or staining and also in regions with crossing fibers. Combined with tomography [32] or 3D-scanning (3D-sSAXS) [33], it can also reconstruct fiber orientations in 3D. However, it requires synchrotron radiation, and the technique can be very time consuming as the tissue needs to be raster-scanned with a pencil beam.

Computational Scattered Light Imaging (ComSLI) overcomes these limitations: Originally developed for nerve fibers [34,35], it has been shown to map all different types of fibers, such as collagen, muscle, or elastin [27,29]. Similar to 3D-PLI, it has micrometer resolution and a large (square-cm) field of view, and does not require expensive equipment (only an LED light source and a camera). However, unlike polarization-based methods, it can reconstruct multiple crossing fiber orientations within a single image pixel, also works on samples with low birefringence (e.g., FFPE brain sections) [26], and is independent of staining [27]. These properties make ComSLI attractive for histological tissue analysis.

ComSLI reverses the SAXS principle: Instead of raster-scanning the tissue with a pencil beam and measuring the scattering pattern (distribution of scattered light) for each location of the beam, the sample is illuminated from many different directions by raster-scanning an illuminated kernel of LEDs across an LED display, and a camera records the normally transmitted (scattered) light of the entire field of view for each illumination direction. In this way, a scattering pattern can be reconstructed from the resulting image series for each micrometer-sized image pixel. The scattering patterns show the distribution of the scattered light and can be used, e.g., to determine the in-plane orientation of the fibers as light scatters mostly perpendicular to the fiber orientation. The resolution of the scattering patterns scales with the number of illumination directions that are sampled, requiring the acquisition of N×N images. As a trade off between measurement time and angular resolution, N=40 is typically chosen [35]. This makes the so-called ComSLI scatterometry relatively time consuming. In many cases, the 2D scattering patterns are not needed as most information such as the in-plane fiber angles can already be obtained from the peak positions in the 1D scattering signals (azimuthal line profiles). In recent years, angular ComSLI, in which the sample is illuminated under a fixed polar angle with a rotating LED spot (requiring only 24 instead of 1600 images or more), established itself as a fast and easy-to-implement technique to accurately determine the in-plane orientation of fibers in various tissue samples [26,27,29,34,36].

Up to now, ComSLI has mostly been used to study two-dimensional fiber orientations: while the in-plane fiber orientations can be accurately determined by analyzing the mid position between scattering peaks, the out-of-plane inclination angles of the fibers could not be quantified so far. However, for accurately reconstructing fiber networks, a good estimate of the 3D-fiber orientations is crucial, e.g., when tracing nerve fiber pathways across consecutive brain sections to generate a 3D-model of the connectome [37,38], but also when computing the angles between collagen fibers and tumor boundaries [27,39]. Prior finite-difference time-domain (FDTD) simulations [40] and experimental studies in anatomically known brain regions [34,36] suggest that inclined fibers yield scattering patterns with a curved high-intensity line, and that the azimuthal distance between scattering peaks, i.e., the azimuthal peak distance in the scattering signals, decreases with increasing inclination angle. However, a functional dependence has not been established yet. To derive an analytical model that describes the curved high-intensity line in a measured scattering pattern and can be used to compute the underlying fiber inclination, and to conclusively validate this model, realistic tissue phantoms with known fiber geometries are needed. However, generating a realistic phantom is challenging: Fibers in biological tissues are often densely packed and interwoven. While skeletal muscle fibers have 40–100 µm diameters [41], nerve fibers in the brain can have diameters of 1 µm or less [42,43,44], and collagen fibrils even down to a few hundred nanometers [45]. Recent advances in Two-Photon Polymerization (2PP) allow to 3D-print phantoms with sub-micrometric feature resolution [46,47], required to approach smaller diameters in biological fiber bundles.

In this study, we performed ComSLI measurements on 3D-nanoprinted phantoms with 200 nm feature resolution that mimic densely grown, biological fiber bundles with different inclinations. The fiber bundles are similar to those used in previous FDTD simulations [40], i.e., they mimic the course of biological fibers as closely as possible (randomly distributed, not completely straight fibers) and the resulting scattering patterns cannot be computed analytically anymore. However, we show here that the highest-intensity points in a measured scattering pattern lie on the same curved line as the diffraction orders of an inclined grating. Using this conical diffraction as analytical model, we were able to estimate the underlying fiber inclinations by fitting conical diffraction curves through the highest-intensity points of the measured scattering patterns. To demonstrate that the conical diffraction model is also valid for biological fibers, we performed ComSLI measurements on a nerve fiber bundle (corpus callosum of a coronal vervet brain section) under different tilt angles, and compared the expected fiber inclinations to those obtained from fitting conical diffraction curves. Finally, we tested the applicability of our analytical model on differently inclined regions of a coronal vervet brain section (corpus callosum, fornix, cingulum). Apart from estimating the fiber inclinations by fitting conical diffraction curves to the measured 2D scattering patterns, we estimated the fiber inclinations from the peak distances of 1D scattering signals that were obtained from an angular ComSLI measurement, allowing us to determine fiber inclinations for each image pixel within the entire field of view. By comparing our results to the inclination angles obtained from a previous 3D-sSAXS measurement of a neighboring vervet brain section, we studied the capabilities and limitations of the technique.

Our findings pave the way towards a quantitative determination of three-dimensional fiber orientations in biological tissue sections, combining ComSLI and advanced light-assisted additive manufacturing.

## 2. Materials and Methods

### 2.1. Inclined Grating Model

The scattering of light is closely related to the geometry of the scatterer so that aligned structures, like nerve fiber bundles, produce anisotropic scattering. Fibers in biological tissue do not form perfect diffraction gratings as they are randomly spaced and not perfectly straight/aligned so that we do not expect to see separate diffraction orders. However, as we show in this study, the directional geometry of the scattered light can be described by conical diffraction at an inclined grating (see Appendix A for more information).

**Figure 1:**
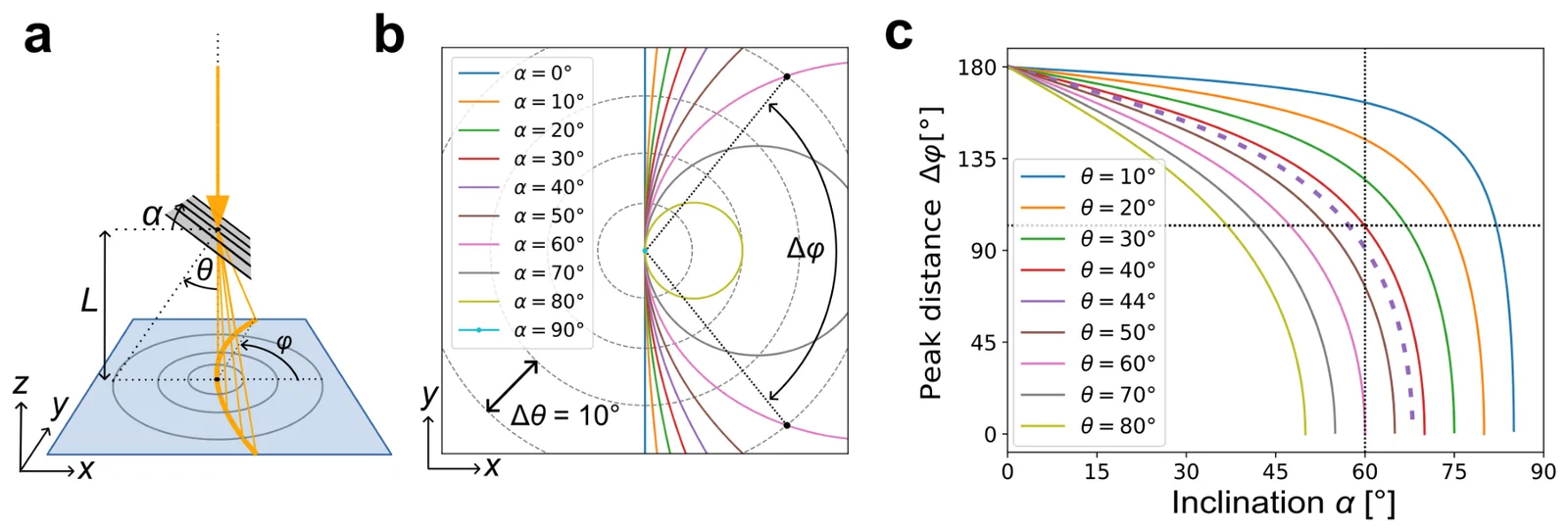
Conical diffraction at an inclined grating. (**a**) Schematic showing the diffraction at a grating with inclination angle α. The orange arrow indicates the direction of the incident light. (**b**) Predicted curves along which the diffraction orders should lie, computed for gratings with different inclinations α. The gray concentric circles indicate polar steps of Δθ=10°. (**c**) Azimuthal peak distance Δφ vs. inclination α for different polar angles θ. The dashed curve indicates the polar illumination angle used in the angular ComSLI measurement. The dotted vertical/horizontal line marks the azimuthal peak distance for θ=40° and α=60° (indicated by Δφ in (**b**)).

When light is incident on an inclined diffraction grating, i.e., under an angle *α* to the grating normal, it produces diffraction orders on the detector screen that lie on a curved line (cf. Figure 1a), and the curvature increases with increasing inclination [48,49,50]. The opening of the curved line is into the direction where the fibers are closest to the detector screen. At the detector plane, the conical diffraction curves are hyperbolas for inclinations 0<|α|<45, and ellipses for |α|>45 (see Figure 1b):

(1) $$y=\pm \frac{\sqrt{xL\sin\left(2\alpha \right)+x^{2}\cos\left(2\alpha \right)}}{\sin\alpha },\;\mathrm{for}\;0<\left|\alpha \right|<90,$$

where (x,y) are the coordinates on the detector screen with (0,0) lying in the center of the screen, and *L* is the distance between the detector screen and the center of the grating. The coordinate system has been chosen such that the *y*-axis lies perpendicular to the grating slits, and the negative *z*-axis in the direction of the incident light (cf. Figure 1a). If the grating slits are parallel to the *x*-axis, i.e., parallel to the screen (α=0°), the diffraction orders lie on a straight line: x=0. If the grating slits are parallel to the *z*-axis, i.e., perpendicular to the screen (α=±90°), the light is not diffracted, i.e., (x,y)=(0,0). The full derivation can be found in Appendix B (Appendix B.1 and Appendix B.2).

When evaluating the conical diffraction curve for a given inclination angle α at a specific polar angle θ, the points of intersection (peaks) lie an azimuthal distance Δφ apart (see Figure 1b). If we know the polar angle θ and the azimuthal peak distance Δφ, we can compute the absolute inclination angle |α| of the grating using the following equation:

(2) $$\left|\alpha \right|=\arctan\left(\frac{\tan(\theta )\cdot \cos(\Delta \varphi /2)}{\sqrt{1+\tan^{2}\left(\theta \right)}-1}\right),\;\mathrm{for}\;0<\theta <90{}^{\circ}.$$

Figure 1c shows a plot of the azimuthal peak distance vs. inclination for different polar angles. The full derivation of Equation (2) can be found in Appendix B.3.

### 2.2. 3D-Printed Phantoms

**Figure A2:**
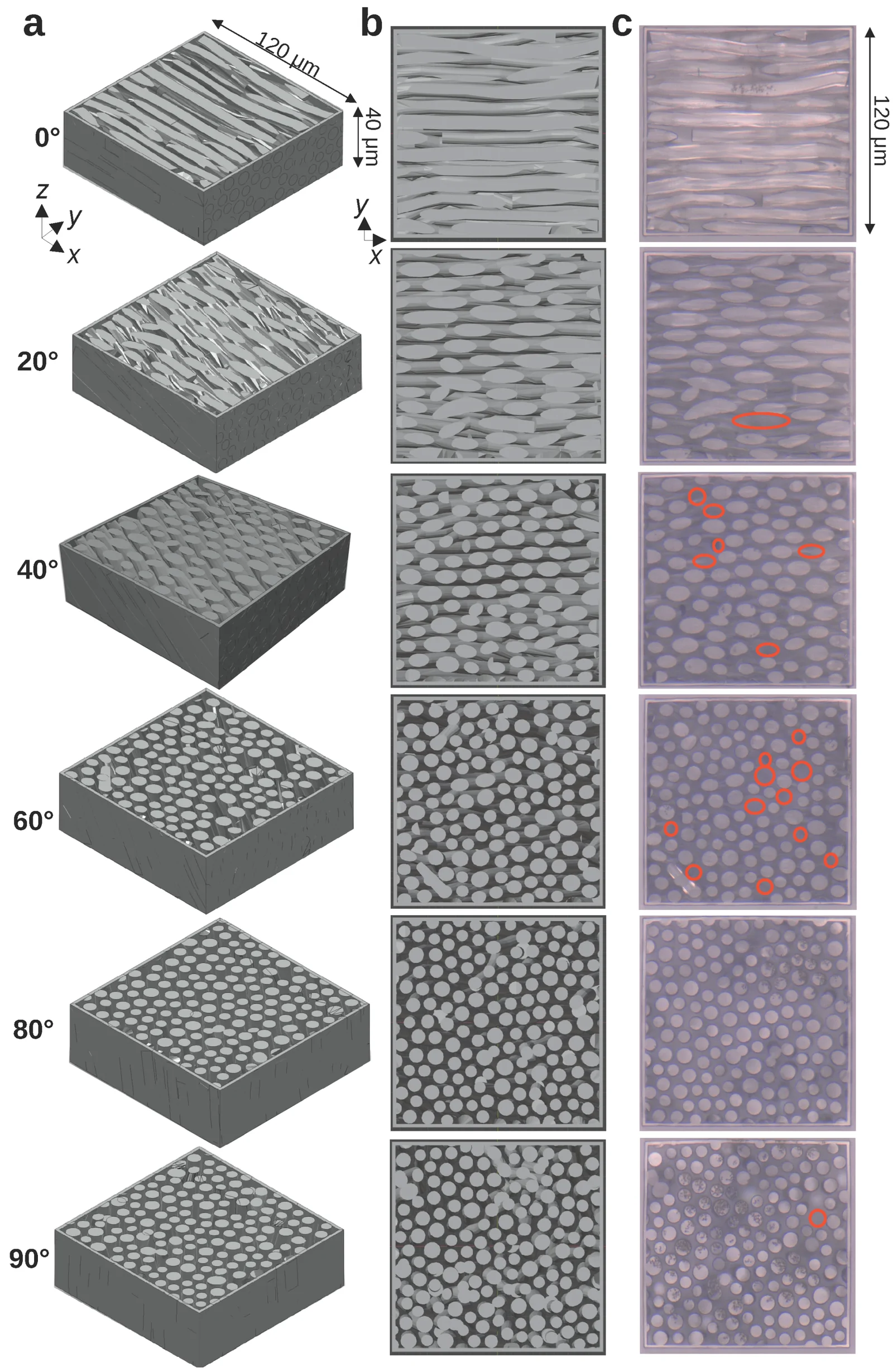
3D-printed fiber bundles (models vs. printed phantoms). (**a**) Rendered 3D-models of the inclined fiber bundles. (**b**) Top-view of the 3D-models. (**c**) Bright-field microscopy images of the 3D-printed phantoms. Areas where fibers are missing are surrounded by red ellipses.

As basis for the 3D-printed phantoms, we used a densely grown fiber bundle to mimic naturally grown fibers in biological tissues. The fiber bundle is similar to the one used in previous simulation studies [40] and was generated with the FastPLI software [51] (version 1.1.3, https://github.com/3d-pli/fastpli, accessed on 28 September 2026), using fiber diameters of (8 ± 1.6) µm. The software starts with a random uniform distribution of points in the (y,z)-plane which are used as starting points for parallel straight fibers along the *x*-direction. Collisions between overlapping fiber segments are solved by slightly displacing the fiber segments, yielding a naturally grown fiber bundle without collisions. The resulting fiber bundle was rotated by different inclination angles (α=0,20,40,60,80,90) and cropped to a volume of 120×120×40 µm^3^. Four side walls with 2 µm thickness were added for stabilization. The resulting 3D-models are shown in Appendix D, Figure A2a.

The three-dimensional fiber phantoms were fabricated by two-photon polymerization using a Photonic Professional GT+ system (Nanoscribe GmbH & Co. KG, Eggenstein-Leopoldshafen, Germany). Two-photon polymerization enables the direct fabrication of three-dimensional microstructures through localized polymerization of a photosensitive material within the focal volume of a tightly focused femtosecond laser beam. Owing to the nonlinear nature of two-photon absorption, polymerization is confined to the focal volume, enabling the fabrication of three-dimensional structures with sub-micrometer features and large overhanging sections without the need for sacrificial support structures.

Before printing, the silica substrates were cleaned and activated with oxygen plasma (Diener electronic GmbH, Ebhausen, Germany) for 30 min at 80 W and an oxygen flow rate of 5 cm^3^/min. The substrates were then treated with undiluted 3-(trimethoxysilyl)propyl methacrylate (MAPTMS) for at least one hour to promote adhesion of the printed fibers. Excess MAPTMS was removed by rinsing the substrates with acetone before printing. The structures were printed on the fused silica substrates using IP-Dip2 (Nanoscribe GmbH & Co. KG, Germany, refractive index n≈1.55 for room temperature and visible light) in DiLL (Dip-in Laser Lithography) mode using a 63× printing objective. Printing parameters were defined using the Describe slicing software and kept constant for all structures. Both the slicing and hatching distances were set to 0.1 µm, resulting in closely spaced voxel trajectories in both the vertical and lateral directions. These parameters were selected to ensure sufficient polymerization throughout the fiber volume while maintaining the designed microscale geometry and minimizing overpolymerization. The laser power was set to 15 mW, and a scan speed of 10,000 µm s^−1^ was used for all structures.

The orientation of the laser scan trajectories relative to the fiber geometry was optimized to facilitate the fabrication of the large unsupported fibers. Specifically, the hatching direction was oriented perpendicular to the longitudinal axis of the fibers, such that individual laser scan trajectories traversed the fiber cross-section rather than extending along the fiber axis. This transverse scanning strategy was employed to improve the structural stability of the fibers during fabrication and reduce the risk of collapse of the unsupported sections.

Following laser exposure, the structures were developed to remove unpolymerized IP-Dip2. Immediately after printing, the substrates were immersed in propylene glycol monomethyl ether acetate (PGMEA) for 25 min, followed by rinsing in isopropyl alcohol (IPA) for 5 min. To minimize capillary forces during drying and thereby reduce the risk of structural collapse, the substrates were subsequently immersed in NOVEC 7100 for 1 min. The substrates were then removed from the solvent and allowed to dry, yielding the final free-standing polymerized fiber structures.

### 2.3. Biological Samples

**Figure A3:**
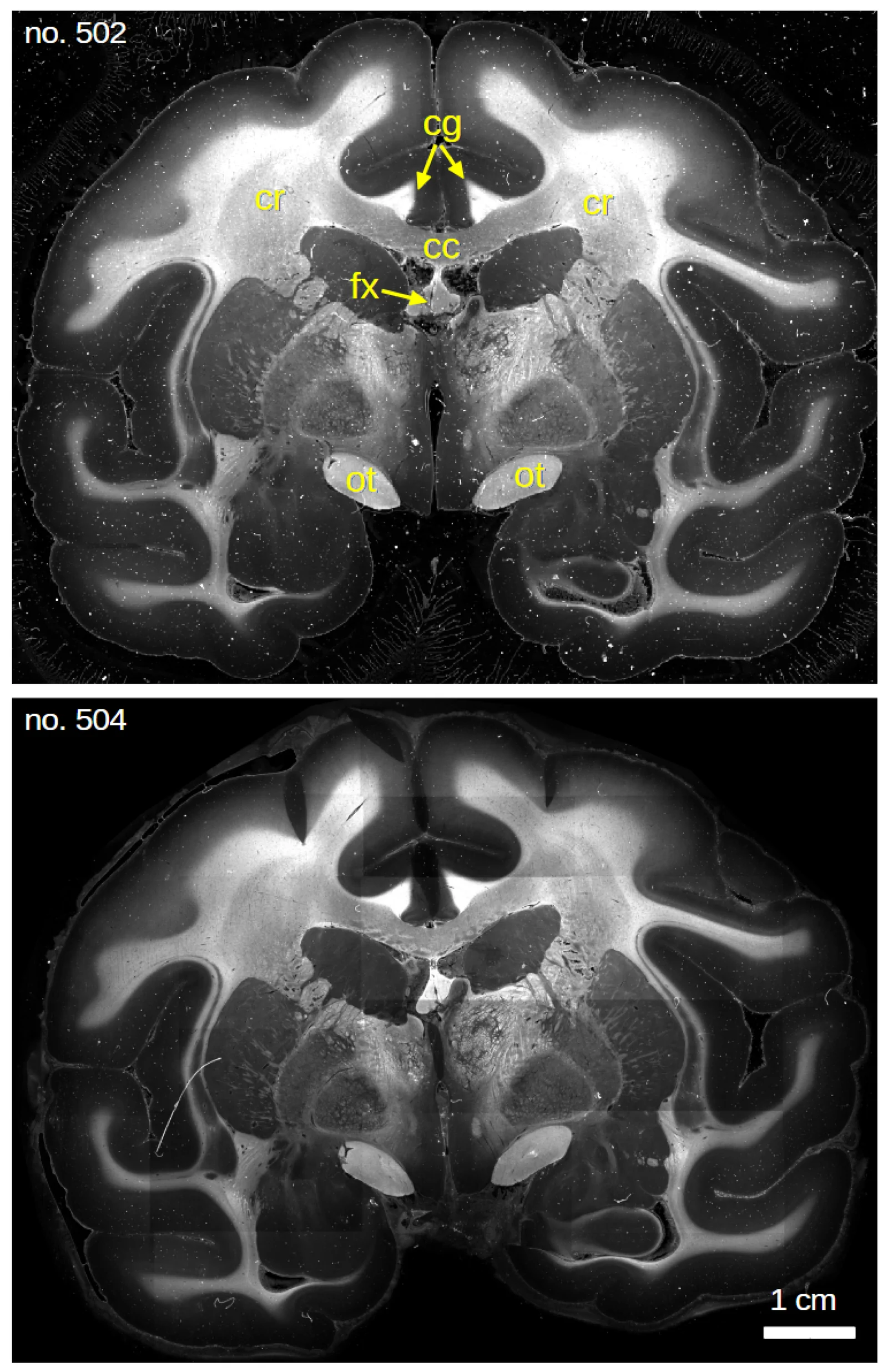
Measured vervet brain sections no. 502 and 504 with anatomical annotations: corpus callosum (cc), fornix (fx), cingulum (cg), corona radiata (cr), optic tract (ot). The images show the averaged scattering signal obtained from angular ComSLI measurements; multiple tiles were stitched together to show the whole brain sections.

For the validation on biological samples, two sections of a vervet monkey brain were used (no. 502 and 504). Appendix D, Figure A3 shows an overview of the samples together with anatomical annotations.

The brain was collected for a different study from a healthy, 2.4-years-old, male vervet monkey. The preparation has been described in previous articles, for example [34,35,36,40]: Following perfusion-fixation with 4% paraformaldehyde, the brain was removed from the skull within 24 h after death and fixed in 4% paraformaldehyde for several weeks. Subsequently, the brain was cryo-protected in 20% glycerin and 2% dimethyl sulfoxide, deeply frozen, and coronally cut from anterior to posterior into 60 µm-thin sections using a cryostat microtome (Polycut CM 3500, Leica Microsystems, Wetzlar, Germany). The resulting brain sections were mounted on glass slides, embedded in 20% glycerin solution, and cover-slipped without staining. Two sections from the middle (no. 502 and 504) were selected for analysis. The embedding took place 14 years before the measurements of this study.

The brain was obtained in accordance with the Wake Forest Institutional Animal Care and Use Committee (IACUC #A11-219). Euthanasia procedures conformed to the AVMA Guidelines for the Euthanasia of Animals, using ketamine/pentobarbital anesthesia followed by perfusion with phosphate buffered saline and fixation with 4% paraformaldehyde. All animal procedures were in accordance with the National Institutes of Health guidelines for the use and care of laboratory animals and in compliance with the ARRIVE guidelines.

### 2.4. Bright-Field Microscopy Measurements

The bright-field microscopy measurements of the 3D-printed phantoms (Appendix D, Figure A2c) were performed with a Keyence VHX-6000 Digital Microscope (Keyence, Osaka, Japan) using a Keyence VH-ZST dual-objective zoom lens with 2000× digital magnification and a high-brightness white LED for illumination.

### 2.5. ComSLI Measurements

#### 2.5.1. Scatterometry Measurement

**Figure 2:**
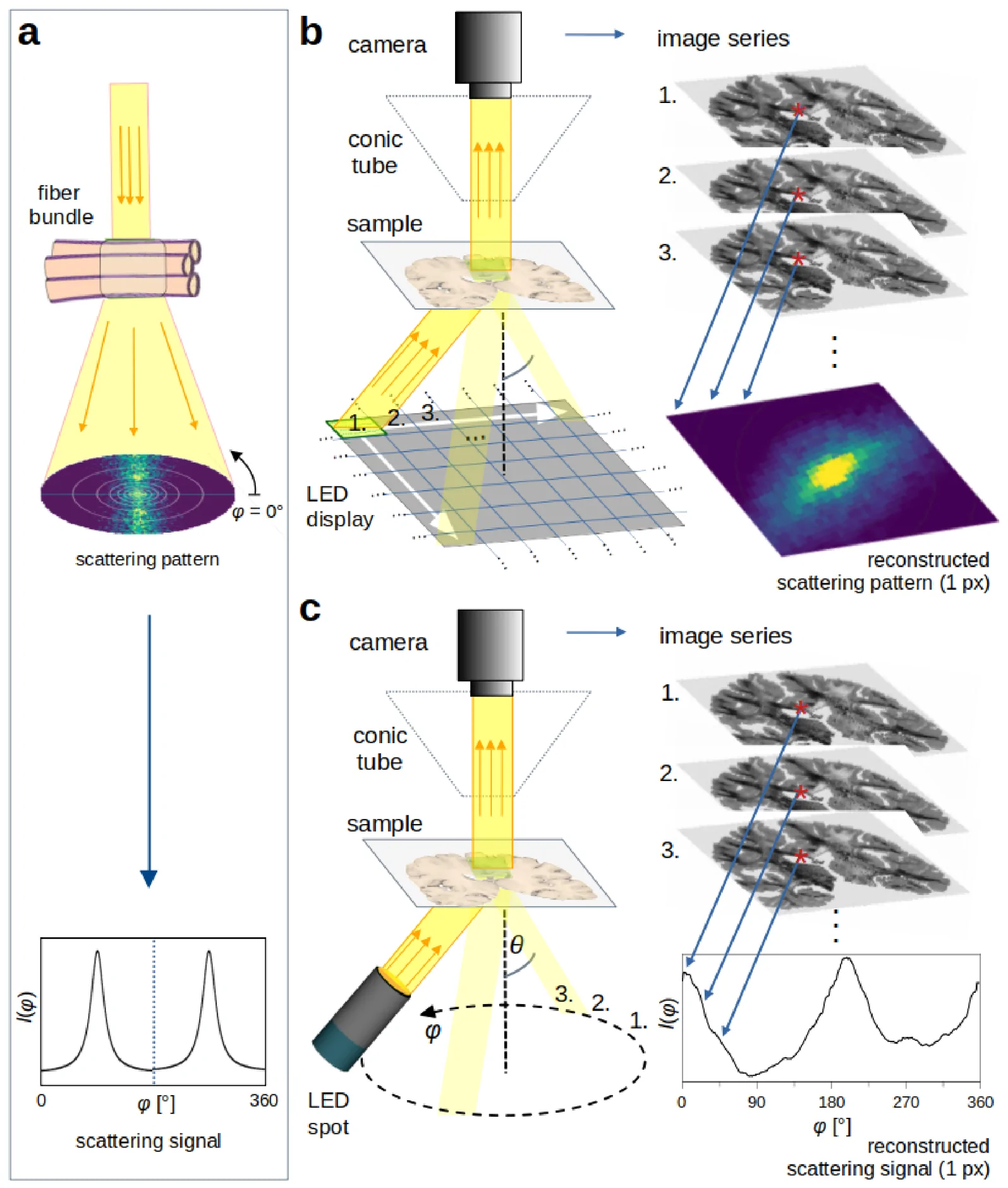
Principle of Computational Scattered Light Imaging (ComSLI) measurements. (**a**) Light scatters perpendicularly to an in-plane fiber bundle, as can be seen in the distribution of the scattered light (scattering pattern, middle). Plotting the scattered intensity as a function of the azimuthal angle φ yields a scattering signal with two distinct peaks (azimuthal line profile I(φ), bottom). (**b**) ComSLI scatterometry measurement. The sample is illuminated by a square of LEDs which is raster-scanned over an LED display in the *x*- and *y*-direction. For every illumination position, the normally scattered light is recorded by a camera. For each pixel in the resulting image series, a scattering pattern can be reconstructed. (**c**) ComSLI angular measurement. The sample is illuminated by a rotating LED spot under a fixed polar angle θ and different azimuthal angles φ. For every illumination angle, the normally scattered light is recorded by a camera. For each pixel in the resulting image series, a scattering signal I(φ) can be reconstructed. One pixel in the image series for which the scattering pattern or the line profile was computed is marked by an asterisk. The top part of this figure has been adapted from Menzel et al. (2023) [36] Figure 1B,D, licensed under CC BY 4.0 (https://creativecommons.org/licenses/by/4.0/). Colors, labels, and scattering patterns have been modified.

The ComSLI scatterometry measurements were performed as described in Menzel et al. (2021b & 2023) [35,36], using the setup shown in Figure 2b, which contains an LED display, a sample stage, and a camera. A light-absorbing conic tube was installed between sample and camera objective to avoid detection of ambient light and suppress internal reflections. For the validation measurements on (inclined) brain sections, a tiltable specimen stage was employed: The stage was attached to the vertical axis via a rotation mount with degree scale; to allow for in-plane rotations, the sample was attached to the rotation mount via an additional joint.

The LED display (Absen LED AW2.8, Absen, Shenzhen, China) contains 176×176 RGB-LEDs arranged in a grid pattern with 2.87 mm pitch. The optical system consists of a CMOS RGB-camera (Basler acA5472-17uc, Basler, Ahrensburg, Germany) and an objective lens (Rodenstock Apo-Rodagon-D120, Rodenstock, Munich, Germany) with 5.6 f-number positioned 241 mm away from the sample. The camera records images with 5472×3648 pixels and a pixel size of approx. 3 µm, yielding a field of view of approx. 1.6 × 1.1 cm^2^. The lateral optical resolution is between 3.9 µm and 4.4 µm (determined by a USAF target).

The samples were illuminated using a 40×40 grid of 2×2 kernels of (white) RGB-LEDs, and an image was taken for every illumination angle. From the resulting image series, scattering patterns were reconstructed for every image pixel (cf. Figure 2b), showing the intensity scattered into the camera for each of the 40×40 illumination positions at a certain location in the sample.

Measurements were performed with gain 1, average 4, and different exposure times to gain enough signal, but avoid over-exposure: 4 s for the 3D-printed phantoms, 3 s for the measurements of the vervet corpus callosum and cingulum, and 2 s for the measurements of the vervet fornix. Samples were placed at different heights above the LED display yielding different polar illumination angles: The 3D-printed phantoms were placed 150 mm above the LED display, yielding a maximum polar illumination angle in *x* and *y* of $\theta_{\mathrm{ill},\max}\approx 37$. Vervet brain section no. 502 (measured under different tilt angles, see Section 3.2) was placed at 255 mm above the LED display ($\theta_{\mathrm{ill},\max}\approx 24$). Vervet brain section no. 504 (measured for different regions, see Section 3.3) was placed at 235 mm above the LED display ($\theta_{\mathrm{ill},\max}\approx 26$). The brain sections were placed higher than the 3D-printed phantoms because the tiltable specimen stage was used for the measurements. As vervet brain section no. 502 had to be rotated in-plane, it was placed slightly higher than the other section to avoid contact with other setup components. For the 3D-printed phantoms, the conic tube was placed such that the opening is 5.6 cm above the sample holder. For measurements with the tiltable specimen stage, the conic tube had to be moved up by 6.5 cm, yielding circular artifacts in the measured scattering patterns. The artifacts were compensated by subtracting the averaged scattering pattern of a region without tissue.

Each scattering pattern has its highest intensity in the center where unscattered light directly reaches the camera. Depending on the position of the image pixel from which the scattering pattern has been generated, the center is at slightly different positions within the 40×40-pixel scattering pattern. When plotting a measured scattering pattern, the center is determined as the mid point of the area with the highest intensity, and concentric rings with Δθ=10° steps are plotted around the center for reference.

#### 2.5.2. Angular Measurement

The angular ComSLI measurements were performed as described in Abbasi et al. (2026) [27], using the setup shown in Figure 2c: Instead of an LED display, the setup contains a rotating LED spot which was rotated at a fixed polar angle (θ≈44°) in Δφ=15° steps around the sample. This step size (24 equidistant illumination angles) is the current standard in angular ComSLI measurements; previous studies [34,35] found this as the best compromise between angular accuracy, measurement time, and data storage amount. Requiring only 24 instead of 1600 illumination angles as for scatterometry measurements reduces the acquisition time from hours to minutes.

The resulting image series was reorganized into scattering signals (azimuthal line profiles) for each image pixel, showing the intensity I(φ) of the normally scattered light recorded by the camera for each of the 24 azimuthal illumination angles φ at a certain location in the sample.

Images were recorded with a monochrome CMOS camera (Basler acA5472-17um). Camera lens and distances were the same as for the scatterometry measurements (Rodenstock Apo-Rodagon-D120 with 5.6 f-number, 241 mm sample-camera-lens distance, 5.6 cm sample-cone distance), yielding the same pixel size, field of view, and resolution. As LED spot, a fiber-coupled white-light LED was used (Prizmatix, Holon, Israel, 400-750 nm range, 443 nm peak wavelength); the light was guided through a 2 m long, multimode fiber with a core diameter of 1500 µm, and passed through a collimator (Prizmatix, FCM1-0.5-CN) and an engineered diffuser (Thorlabs ED1-S20-MD, Thorlabs, Newton, NJ, United States) for beam shaping.

All measurements were performed with an exposure time of 15 ms, averaging over 4 images, and 130 mm distance between sample and LED spot.

### 2.6. 3D-sSAXS Measurement

The 3D-scanning small-angle X-ray (3D-sSAXS) measurement (used for validating the determined fiber inclinations in vervet brain section no. 504) has been performed on a neighboring vervet brain section (section no. 511, prepared as described in Section 2.3) for a different study (Menzel et al. (2023) [36]). For the 3D-sSAXS measurement, the brain section was removed from the glass slides, immersed in phosphate-buffered saline solution for two weeks, placed between two 170 µm-thin cover slips, and sealed. The measurement was performed with a beam of 15 keV photon energy, 100 µm diameter, 0.7 s exposure time, (0,±15°,…,±60°) rotation angles, 19.0 × 10.9 mm^2^ field of view, and 100 µm-steps in *x* and *y*. The fiber inclination angles were computed by analyzing the scattering patterns at different sample rotation angles as described in Georgiadis et al. (2020) [33]. The fiber inclination angles are shown in Figure 6A in [36], and the dataset [52] is available from Zenodo under a CC BY 4.0 license (https://creativecommons.org/licenses/by/4.0/); histograms were generated for selected regions for comparison.

### 2.7. Data Analysis

#### 2.7.1. Conical Diffraction Curve Fitting

**Figure A4:**
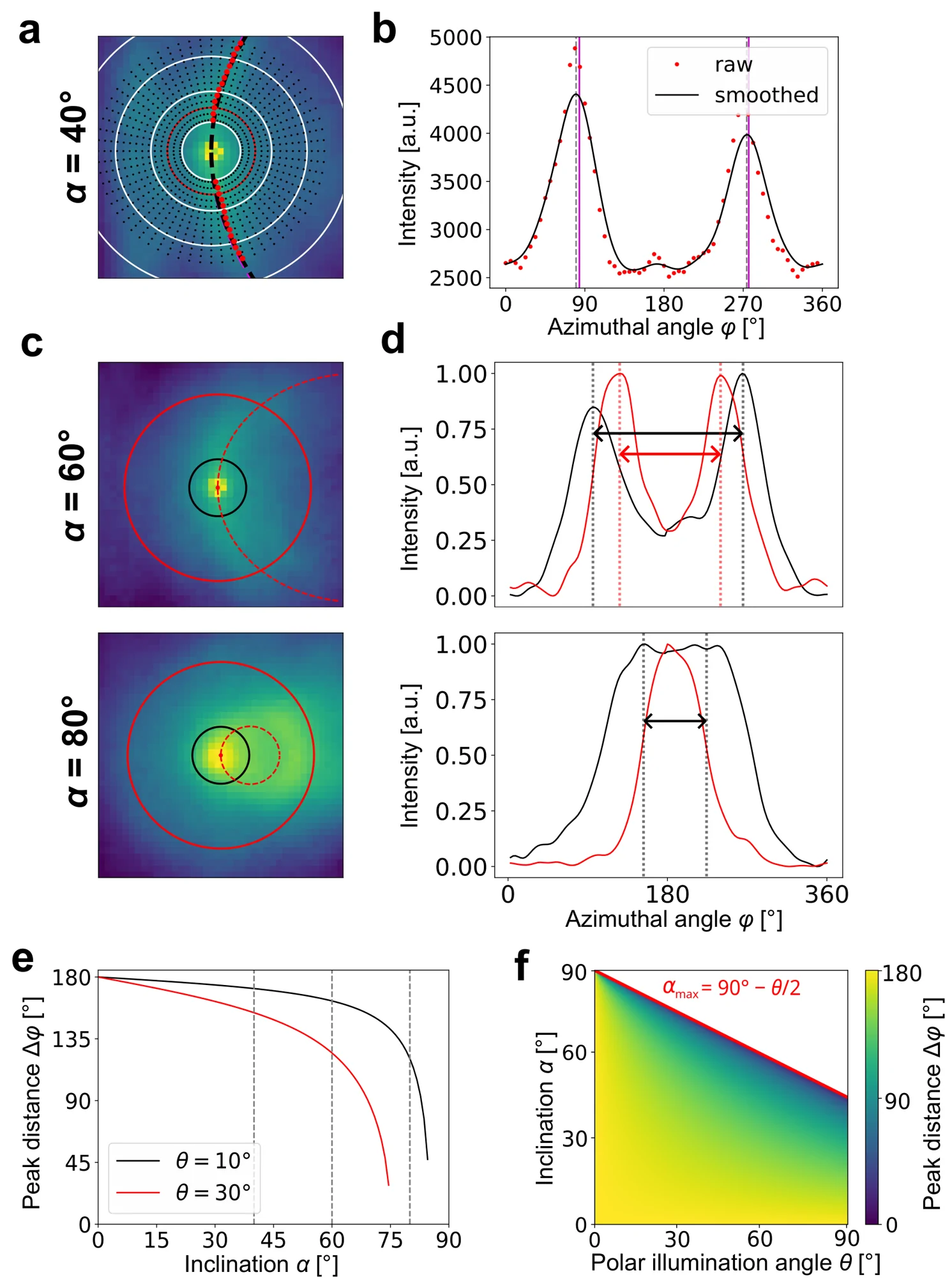
Radial peak finding and peak distance of azimuthal line profiles. (**a**,**b**) Example scattering pattern and azimuthal line profile (for 40°-inclined 3D-phantom and θ=15°). The black dots in (**a**) indicate the points that were evaluated to identify the points of highest intensity (red dots) for each azimuthal line profile. The graph in (**b**) shows both the raw data (red points) and the smoothed profile (black line) that was used to determine the peaks (black dashed lines). The magenta lines indicate the theoretical curve. (**c**) Scattering patterns of the 3D-printed fiber bundles for different inclination angles α. The rings with θ=10° and θ=30° are marked by black and red circles, respectively, and the theoretical curves by red dashed lines. (**d**) Corresponding azimuthal line profiles for θ=10° (black) and θ=30° (red). The double-headed arrows indicate peak distances. (**e**) Azimuthal peak distance Δφ vs. inclination α for θ=10° (black) and θ=30° (red). The inclination angles of the top part of the figure (α=40,60,80°) are indicated by vertical dashed lines. (**f**) Inclination α vs. polar illumination angle θ for different peak distances Δφ (colors). The red line indicates the maximum possible inclination angle $\alpha_{\max}$ that can be computed from an angular ComSLI measurement with polar illumination angle θ.

To estimate the fiber inclination angle from a measured scattering pattern, the conical diffraction curves (Figure 1b) were fitted to the points of highest intensity in the scattering patterns, not taking the unscattered light in the center into account. To identify the points of highest intensity, a peak finding approach was used as illustrated in Appendix D, Figure A4a,b:

Azimuthal line profiles were calculated from a scattering pattern for circles with radii between 5 and 18 pixels in steps of 1 pixel. 5 and 18 pixels are the minimum and maximum possible radius within a 40 × 40 px scattering pattern, excluding the unscattered light in the center and taking a maximum center shift of 2 pixels into account. The profiles show the interpolated intensity of the scattering pattern at 64 equidistant azimuthal angles for each radius (see Figure A4a, black dots). The azimuthal profiles were sub-sampled in 1°-steps and subsequently smoothed by a Gaussian kernel with a standard deviation of 12° (Figure A4b). In the resulting smoothed profiles, peak positions were determined using the scipy.signal.find_peaks function of the SciPy Python library (version 1.15.3) [53], using a repeated version of the signal to take the periodicity into account. Peaks were ignored if they were less than 50% of the maximum of the signal, or if their prominence was less than 0.1% of the maximum of the signal. The prominence is defined by the vertical distance between the top of the peak and the higher of the two neighboring minima, and it was determined using the scipy.signal.peak_prominences function. The two peaks with the highest prominence were selected and marked in the scattering pattern as red dots (see Figure A4a).

Finally, a least-squares fit was used to find the theoretical diffraction curve that most closely matches these points of highest intensity, using (Δφ,Δα)=0.1°-steps. Only curves going through the center of the scattering pattern were considered. For the 3D-printed phantoms, an in-plane angle of φ=0° was assumed. For the biological samples, both α and φ were fitted. To estimate the goodness of the fitted inclination and in-plane angle, the root-mean-square (RMS) distance was computed for all different inclinations and in-plane angles for the fitted φ- and α-values, respectively, and the range of inclinations and in-plane angles for which the RMS distance differs <1% from the minimum was determined.

#### 2.7.2. Fiber Inclination from Peak Distance

The angular ComSLI measurements were analyzed as described in Menzel et al. (2021a) [34], using the Scattered Light Imaging ToolboX (SLIX), version 2.4.2 (https://github.com/3d-pli/SLIX, accessed on 28 September 2026) [54]. SLIX has been optimized for analyzing angular ComSLI measurements with 15°-azimuthal steps, and shown to reliably reconstruct (in-plane) fiber orientations per image pixel with 2.5° standard deviation (accounting for the 15°-discretization of the line profiles by computing the geometric center of the peak tip [34]). Apart from an in-plane fiber orientation map, SLIX computes the azimuthal peak distance Δφ (distance between two prominent peaks in the azimuthal line profile) for every image pixel. Peaks with a prominence above 8% of the total signal amplitude (max − min) were considered as prominent peaks. Pixels with one peak have zero peak distance and pixels with more than two peaks were not evaluated as these are considered to contain crossing fibers or belong to noisy signals.

The inclination angle α was computed from the peak distance Δφ, using Equation (2) with polar illumination angle θ=44° (dashed curve in Figure 1c). Pixels with zero peak distance were here attributed an inclination angle of 68°, meaning that higher inclination angles could not be determined for these pixels.

## 3. Results

We first analyzed the scattering patterns of samples with known fiber orientations. For this purpose, we performed ComSLI scatterometry measurements on the 3D-printed phantoms with different fiber inclinations, and fitted the conical diffraction curves to the measured scattering patterns (Section 3.1). Subsequently, we compared our results to measurements of biological fibers (nerve fibers in the vervet brain sections). To study nerve fibers with different inclination angles, we selected a region with mostly in-plane parallel fibers, tilted the brain section by different angles, and analyzed the resulting scattering patterns (Section 3.2). Finally, we measured different regions in a vervet brain section with ComSLI scatterometry and estimated the fiber inclinations from the measured scattering patterns (Section 3.3). To investigate the potential of speeding up the measurement, we performed an angular ComSLI measurement on the same brain section, estimated the local fiber inclinations from the azimuthal peak distances, and compared our results to the fiber inclinations obtained from a 3D-sSAXS measurement of a neighboring section.

### 3.1. 3D-Printed Phantoms

To verify that the 3D-printed phantoms resemble the original 3D-models of which we know the fiber geometries/inclinations, we measured all 3D-printed phantoms with a bright-field microscope (Appendix D, Figure A2c). Comparing these images to the original 3D-models (Figure A2a,b) shows that a few of the 3D-printed fibers are missing for intermediate inclination angles (marked by red ellipses). These fibers detached from the fused silica substrate during development, likely due to imperfect adhesion, swelling, or small bubbles in the resin. Nevertheless, the positions and shapes of all printed fibers correspond to what we would expect from the original 3D-model. Therefore, we can assume that the printed fibers are oriented as defined by the 3D-models, i.e., all fibers have an average in-plane azimuthal angle of φ=0° (along the *x*-axis) and an average out-of-plane inclination angle of α={0,20,40,60,80}°.

**Figure 3:**
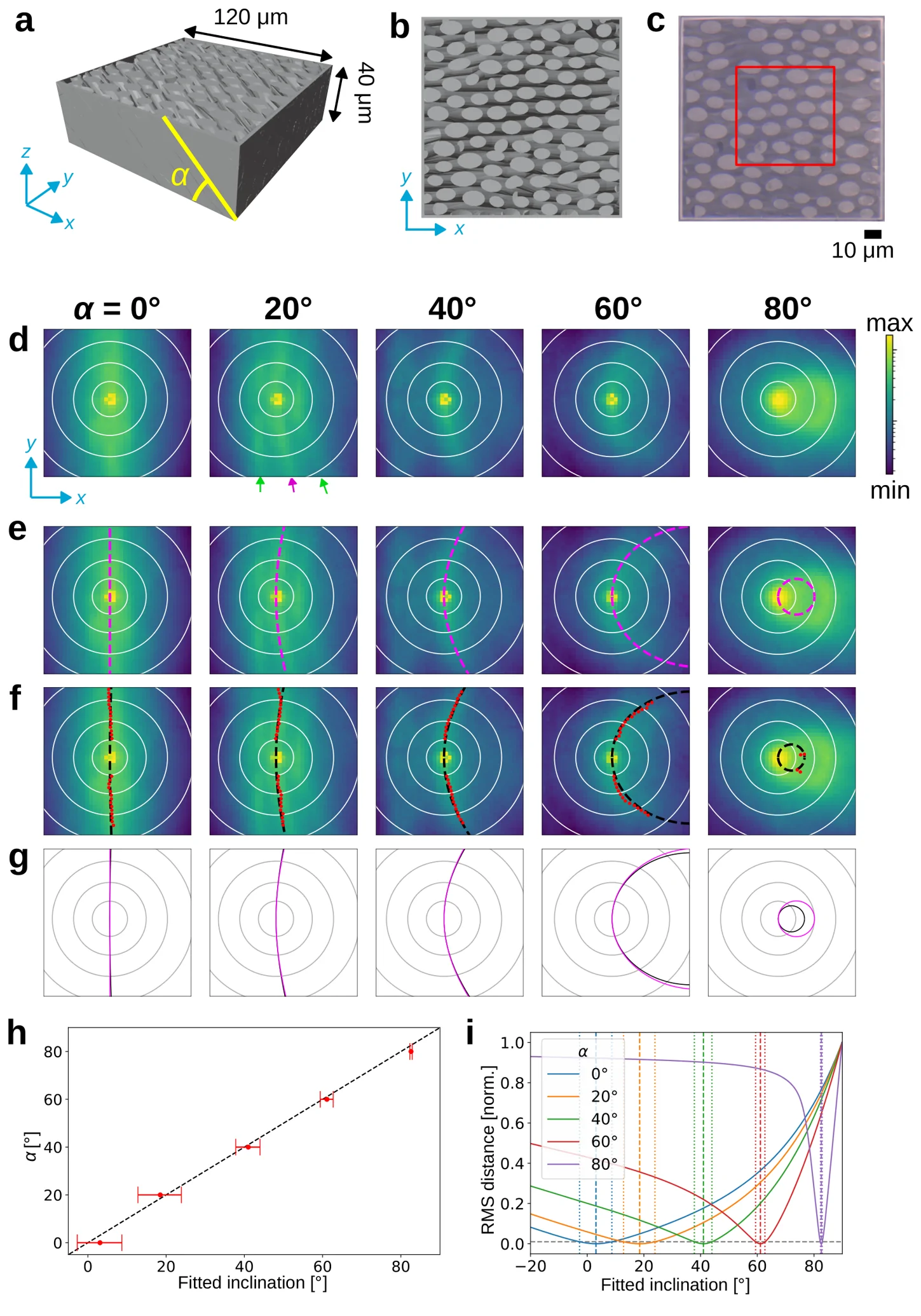
Scattering patterns of 3D-printed fiber bundles for different inclination angles. (**a**) 3D-model, shown exemplary for α=40°. (**b**) Top view of the 3D-model. (**c**) Bright-field microscopy image of the 3D-printed phantom. The red square indicates the region in which the scattering patterns were evaluated. The scale bar is valid for both (**b**,**c**). (**d**) Scattering patterns for different inclination angles, averaged over 20×20 image pixels (red square in (**c**)). White circles indicate Δθ=10° steps. For the α=20° phantom, green arrows indicate side lines, while the magenta arrow indicates the middle high-intensity line going through the center. (**e**) Scattering patterns with theoretical curves (magenta). (**f**) Scattering patterns with highest-intensity points (red) and fitted curves (black). (**g**) Comparison between theoretical curves (magenta) and fitted curves (black). (**h**) Fiber inclination α vs. fitted inclination (including 1% RMS distance intervals). The dashed line is the theoretical expectation. (**i**) Normalized root-mean-square (RMS) distance between the fitted lines and the highest-intensity points for the differently inclined fiber bundles, as a function of the fitted inclination. The dotted vertical lines indicate the ranges for which the RMS distance differs <1% from the minimum. The values for the fitted inclination, 1% RMS distance intervals, minimum RMS distance and standard deviation are listed in Appendix C, Table A1.

Figure 3 shows the results of the ComSLI scatterometry measurement for the differently inclined 3D-printed fiber bundles, and the comparison with the conical diffraction model. To obtain a representative scattering pattern for each 3D-printed phantom, the scattering patterns obtained from the inner 20×20 image pixels of each sample were averaged. Figure 3a–c shows the 3D-model, top-view, and microscopy image exemplary for the 40°-inclined phantom; the red square in (c) indicates the region in which the scattering patterns were evaluated.

The resulting averaged scattering patterns are displayed in Figure 3d. They show a high-intensity line going through the center; its curvature increases with increasing inclination and the opening is towards the right, i.e., where the fibers are closest to the LED-display. For some patterns, additional side lines are visible, indicated by green arrows for the 20° phantom. The middle high-intensity lines closely match the theoretical conical diffraction lines that were computed for the respective inclination angles α (magenta dashed lines in Figure 3e).

To check whether the theoretical curves accurately describe the measurement data and to investigate how reliably we can estimate the fiber inclination from a measured scattering pattern, we fitted the conical diffraction model to the measured scattering patterns (Figure 3f). The red dots show the highest-intensity points determined from the peak finding approach, and the black dashed lines show the curves obtained from the least-squares fit as described in Section 2.7.1. Figure 3g shows a direct comparison of the fitted curves (black) and theoretical curves (magenta), demonstrating that the fitted curves closely match the theoretical diffraction curves, especially for α≤40°.

In Figure 3h, we see the inclination angles of the 3D-printed fibers that were used to compute the theoretical diffraction curves plotted against the inclination angles estimated from the fitting (red dots). The estimated inclination angles lie close to the theoretical expectation (diagonal dashed line); the maximum deviation is 3.1° for the in-plane fiber bundle. The graph in Figure 3i shows the normalized RMS distance between the highest-intensity points and the fitted line for different (fitted) inclination angles. To indicate the precision of our fit, the dotted vertical lines mark the ranges for which the RMS distance differs less than 1% from the minimum; these ranges are also indicated in (h). The exact values (fitted inclination angles, 1% RMS distance intervals, minimum RMS distance and standard deviation) are listed in Appendix C, Table A1. For α={0,20,40,60}°, the inclination angles estimated from the fitting correspond to the actual fiber inclination angles of the 3D-models within the 1% RMS distance ranges. For α=80°, the estimated inclination is slightly over-estimated, even when taking this range into account.

As can be seen in Figure 3i, the RMS distance vs. fitted inclination curve has a broader minimum and thus a lower precision for smaller inclination angles than for larger inclination angles. This is due to the fact that the theoretical diffraction curves for smaller inclination angles lie closer together than for larger inclination angles (cf. also Figure 1b). Even if the highest-intensity points lie almost perfectly on a conical diffraction curve for a certain α, another diffraction curve with a slightly smaller or larger α describes these points almost equally well so that a slight change in the points’ locations already leads to a different fitted inclination: while the theoretical/fitted curves in Figure 3g look almost identical for α=0°, the estimated inclination differs by 3.1° from the theoretical value. For larger inclination angles, the fitting is much more stable: while the theoretical/fitted curves in Figure 3g look clearly different for α=80°, the estimated inclination only differs by 2.6° from the theoretical value.

However, larger inclination angles have another problem: Instead of a clearly defined high-intensity line/hyperbola, the highest intensity is expected along an ellipse, leading to an accumulation of intensity and making it more difficult to accurately determine the points of highest intensity. As can be seen in Figure 3f, only four points of highest intensity were found for α=80° while the right part of the high-intensity region does not yield clearly defined intensity peaks. This leads to a wrong fitting and thus an over-estimation of the actual inclination angle. Although the 1% RMS distance intervals are very small for large inclinations, the fitted inclination is therefore likely to be inaccurate.

Despite these challenges, all fitted inclination angles were found to be mostly accurate up to ±3°, showing that the conical diffraction model accurately describes the measurement data and can be used to estimate the underlying fiber inclination.

### 3.2. Tilted Vervet Brain Section

So far, we have only considered artificial fibers. To find out whether the conical diffraction model can be used to estimate the inclination angles of biological fibers, we investigated differently inclined nerve fiber bundles in a vervet monkey brain section. Neighboring sections from the same vervet brain have already been used in multiple studies, for example [34,35,36,40], to investigate nerve fiber orientations with scattered light imaging.

**Figure 4:**
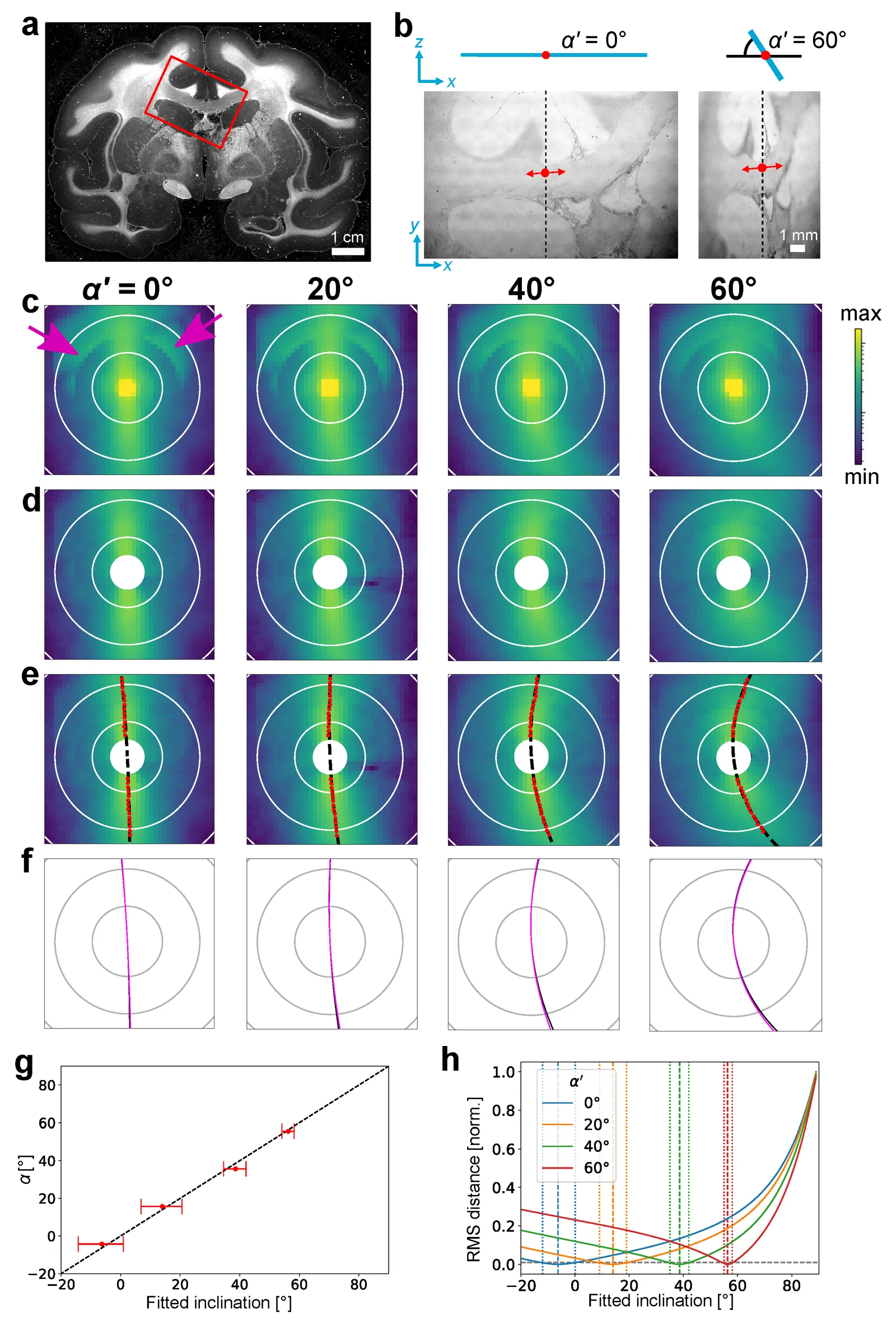
Scattering patterns of a nerve fiber bundle (vervet corpus callosum) for different tilt angles $\alpha^{\prime }$. (**a**) Overview image of the vervet brain section (averaged scattering intensity of section no. 502). The red rectangle marks the region shown in (**b**). (**b**) Images of the sample during measurements with 0° and 60° tilt angle. The dashed vertical lines indicate the tilting axis. The scattering patterns were evaluated at the location indicated by the red dot, where fibers are mostly oriented horizontally (double-headed arrows). (**c**) Scattering patterns obtained for $\alpha^{\prime }=\{0,20,40,60\}{}^{\circ}$ tilt angles (averaged over 20×20 image pixels, indicated by the red dot in (**b**)). White circles indicate Δθ=10° steps. Circular artifacts are marked by magenta arrows. (**d**) Calibrated scattering patterns. The center is not shown as it was not considered for the fitting. (**e**) Calibrated scattering patterns with highest-intensity points (red) and fitted curves (black). (**f**) Comparison between fitted curves (black) and theoretical curves (magenta). For the theoretical curves (theoretical α,φ-values), it was assumed that the zero-tilt fiber orientation corresponds to the average of the fitted (α,φ)-values, back-rotated to their orientation before the tilt. (**g**) Theoretical fiber inclination α vs. fitted inclination (including 1% RMS distance intervals). The dashed line is the theoretical expectation. (**h**) Normalized root-mean-square (RMS) distance between the fitted line and the highest-intensity points for the different tilt angles, as a function of fitted inclination. The dotted vertical lines indicate the ranges for which the RMS distance differs <1% from the minimum. The fitted inclination and in-plane angles, the corresponding 1% RMS distance intervals, the minimum RMS distance and standard deviation, as well as the theoretical α,φ values are listed in Appendix C, Table A2 and Table A3.

To study nerve fiber bundles with different inclination angles, we selected a region with mostly in-plane, horizontal fibers (corpus callosum in a coronal brain section) and tilted the section by different tilt angles to mimic different fiber inclinations. Figure 4 shows the results of the ComSLI scatterometry measurements and the comparison to the conical diffraction model for different tilt angles.

Vervet brain section no. 502 (Figure 4a) was selected for the analysis. The section was rotated in the (x,y)-plane to align the fibers in the corpus callosum with the horizontal *x*-axis, and tilted around the *y*-axis by different angles to mimic different out-of-plane fiber inclinations (see Figure 4b). The horizontal alignment was required to ensure that the in-plane orientations of the fibers do not change much during the tilting and that the out-of-plane angles increase mostly linearly with the tilt angle. For evaluation, we selected a (20×20)-pixel region that lies on the tilt axis (indicated by the red dots in Figure 4b) to ensure that the evaluated regions lies in focus for all tilt angles. The section was tilted by $\alpha^{\prime }=\{0,20,40,60\}{}^{\circ}$ to achieve similar fiber inclinations as the ones in the 3D-printed phantoms; larger tilt angles were not possible as the tiltable specimen stage gets too close to the LED display and hinders the measurement. As mentioned in Section 2.5.1, the conic tube had to be moved up for the tilting, leading to circular artifacts in the averaged scattering patterns (Figure 4c, magenta arrows). To remove the circular artifacts, the original scattering patterns were calibrated by subtracting the averaged scattering pattern of a region without tissue for the respective tilt angle (Figure 4d).

Figure 4e shows the highest-intensity points of the calibrated scattering patterns (red dots) determined by the peak finding approach together with the fitted conical diffraction curves (black dashed lines). In contrast to the 3D-printed phantoms for which the underlying fiber orientations and thus the theoretically expected diffraction curves are known, the nerve fiber orientations in the measured region are not exactly known. Although we selected a region in the corpus callosum which is known to contain mostly parallel in-plane fibers in a coronal brain section [55,56], the fibers could still have a small out-of-plane inclination angle which can be positive/clockwise or negative/counter-clockwise. Also the in-plane fiber angle can slightly differ from zero as the microscopic fiber orientations are not visible and the fiber bundle can only be roughly aligned with the *x*-axis of the specimen stage. To still investigate whether the fitted fiber orientations are self-consistent taking the respective tilt angles into account, we fitted both fiber inclination and in-plane angle for each tilt angle, rotated the fitted fiber orientation ($\varphi_{\mathrm{fit}},\alpha_{\mathrm{fit}}$) back to the orientation before the tilt, and took the average over all tilt angles as theoretical expectation for zero-tilt fibers: $\left(\varphi_{\mathrm{theory}},\alpha_{\mathrm{theory}}\right)=\left(3.0{}^{\circ},-4.3{}^{\circ}\right)$. Figure 4f shows the fitted curves (black) in comparison to the theoretical curves (magenta). Figure 4g,h show the theoretical inclination and normalized RMS distance plotted against the fitted inclination together with the respective 1% RMS distance intervals. The exact values (fitted and theoretical inclination/in-plane angles, back-rotated values, 1% RMSD intervals, minimum RMS distance and standard deviation) can be found in Appendix C, Table A2 and Table A3.

As can be seen, the fitted curves closely match the theoretically expected diffraction curves, and the fitted inclination values correspond to the theoretical inclination values within their 1% RMS distance intervals. The difference between fitted and theoretical inclination angles lies between 0.7° for $\alpha^{\prime }=60{}^{\circ}$ and 3.0° for $\alpha^{\prime }=40{}^{\circ}$. The difference between fitted and theoretical in-plane angles lies between 0.2° for $\alpha^{\prime }=0{}^{\circ}$ and 0.8° for $\alpha^{\prime }=60{}^{\circ}$.

This shows that the conical diffraction model also accurately describes the scattering on biological fibers such as nerve fibers, at least until an inclination angle of about 60°. Larger inclination angles could not be validated with the employed tiltable specimen stage.

### 3.3. Different Vervet Brain Regions

**Figure 5:**
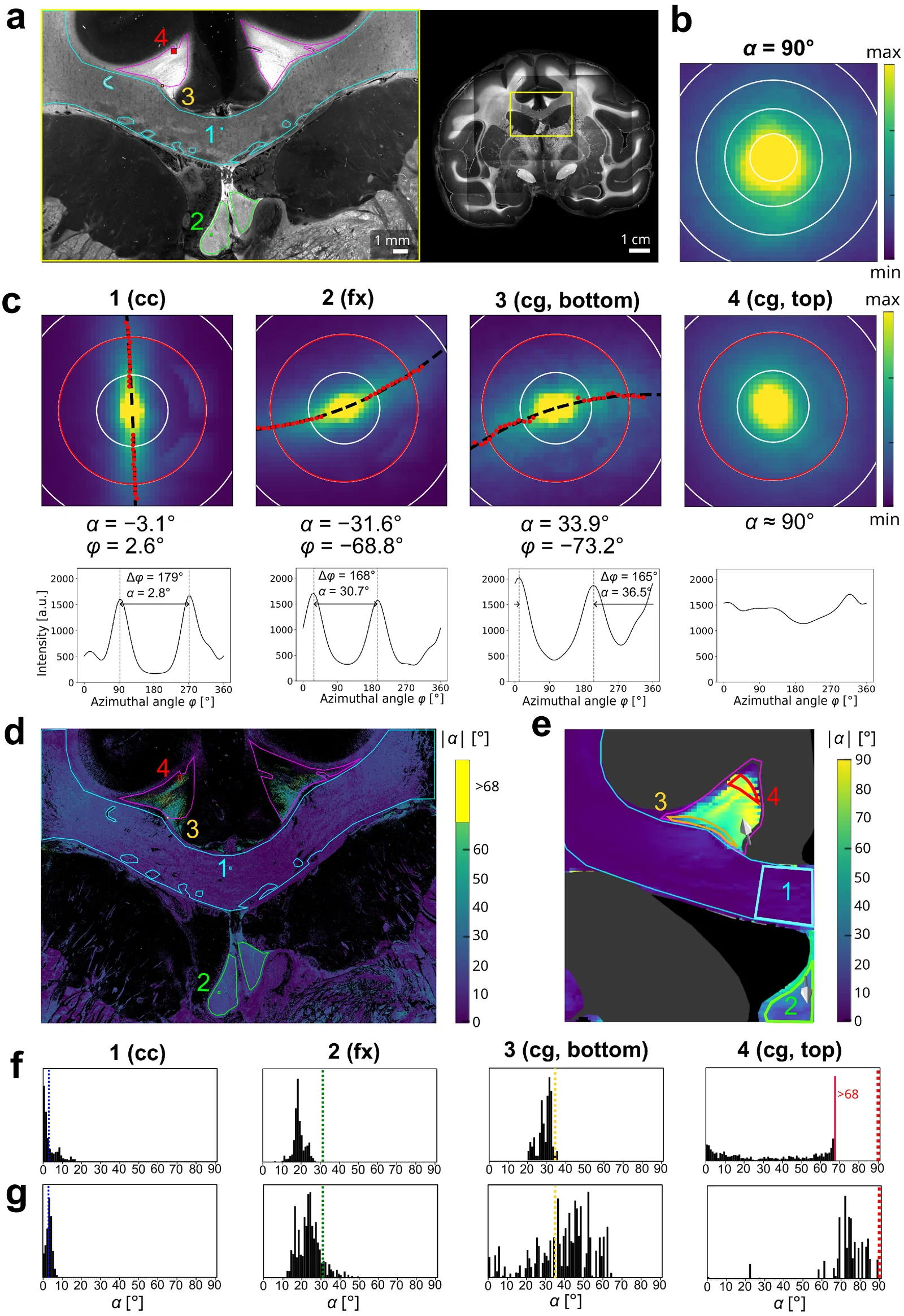
Fiber inclinations of different vervet brain regions estimated from scattering patterns and azimuthal peak distances. Vervet brain section no. 504 was used for the measurements. (**a**) Right: Overview image of the vervet brain section (averaged scattering signal). Left: Zoom-in of the region for which the azimuthal peak distances were determined (marked by the yellow rectangle). The colored squares 1, 2, 3 and 4 indicate the locations in the corpus callosum (cc—25×25 px), fornix (fx—25×25 px), bottom cingulum (cg, bottom—10×10 px) and top cingulum (cg, top—50×50 px), respectively, that were used to compute the scattering patterns. The larger surrounded white-matter regions were used to calculate the number of peaks in Table 1. (**b**) Scattering pattern of the 3D-printed phantom with vertical fibers (α=90°), used for comparison. (**c**) Scattering patterns of the four regions with highest-intensity points (red dots), fitted curves (black dashed lines), fitted fiber inclinations α, and fitted in-plane angles φ. The 1% RMS distance intervals, minimum and standard deviation of the root-mean-square distances are listed in Appendix C, Table A4 and Table A5. The bottom graphs show the azimuthal line profiles I(φ) at θ=20° (red circles) together with the azimuthal peak distances Δφ and the corresponding estimated inclinations α. The arrows in the line profiles indicate the peak distance Δφ. (**d**) Inclination map generated from an angular ComSLI measurement with θ=44°, with the regions from (**a**) indicated. (**e**) Inclination map generated from a 3D-scanning small-angle X-ray scattering (3D-sSAXS) measurement of a neighboring vervet brain section [52] (section no. 511, shown in Figure 6A in [36], used under CC BY 4.0 (https://creativecommons.org/licenses/by/4.0/)). Annotations were removed and the image was cropped and mirrored at the *y*-axis as the brain section was measured from the other side. 3D-arrows were added to indicate the out-of-plane fiber directions in fornix and cingulum (after mirroring). Four regions (labeled 1–4) were selected for evaluation. (**f**,**g**) show histograms of the inclinations from the angular ComSLI and 3D-sSAXS measurements, respectively, for the four marked regions. The colored vertical lines indicate the fitted inclination values obtained from the measured scattering patterns.

Next, we estimated the fiber inclinations for different brain regions from the measured scattering patterns (scatterometry ComSLI) as well as from the azimuthal line profiles (angular ComSLI). The results are shown in Figure 5.

Scattering patterns were obtained for four different regions in vervet brain section no. 504 (Figure 5a): 1–corpus callosum (cc), 2–fornix (fx), 3–bottom cingulum (cg, bottom), and 4–top cingulum (cg, top). Figure 5c shows the (averaged) scattering patterns of these regions together with the fitted conical diffraction curves (black dashed lines). The corpus callosum and fornix were averaged over 25×25 patterns, while the bottom cingulum was averaged over 10×10 patterns as this was the largest homogeneous region that could be found in this area. The top cingulum was averaged over 50×50 patterns to average out speckle that was observed in these patterns. The fitted inclination and in-plane angles (α,φ) are indicated below the patterns. The graphs at the bottom show the azimuthal line profiles evaluated at 20° polar angle (red circles in the scattering patterns).

As expected, the corpus callosum contains mostly in-plane fibers (α=−3°) and the azimuthal line profile shows two clear peaks that lie almost 180° apart. The fornix (α=−32°) and especially the cingulum (α=34°) contain more out-of-plane fibers; the azimuthal peak distances Δφ are smaller than for the corpus callosum. The top part of the cingulum (region 4) yields a mostly isotropic scattering pattern with no high-intensity line and an almost flat azimuthal line profile, indicating a highly inclined fiber bundle. Comparing this scattering pattern to the one obtained from a 3D-nanoprinted phantom with α=90° (Figure 5b) shows that this region contains probably nearly vertical fibers.

Measuring the (40×40)-pixel scattering patterns with a ComSLI scatterometry measurement (requiring 1600 images) and computing the fitted inclination angles from each scattering pattern is very time consuming. To investigate whether we can speed up the measurement and estimate the inclination angles of all image pixels in a larger field of view, an angular ComSLI measurement was performed on the same brain section, in the region containing corpus callosum, fornix, and cingulum (yellow rectangle in Figure 5a) using a polar illumination angle of θ≈44° (see Section 2.5.2). The inclination angles were computed from the azimuthal peak distances as described in Section 2.7.2. The resulting inclination map is shown in Figure 5d and histograms of the four square regions (1–4) from which the scattering patterns were computed are shown in Figure 5f (with the fitted inclination angles indicated by colored vertical lines).

To validate the fiber inclinations obtained for vervet brain section no. 504, we compared the inclinations to those obtained from a 3D-sSAXS measurement of a neighboring section (see Section 2.6) that was measured for a previous study [52] (section no. 511, shown in Figure 6A in [36]). 3D-sSAXS has been shown to reliably determine the 3D-orientations of nerve fibers in brain tissue [33]. Figure 5e shows the inclination map from the 3D-sSAXS measurement and Figure 5g shows the histograms for areas 1–4 marked in (e). As the sections used for angular ComSLI (no. 504) and 3D-sSAXS (no. 511) lie 0.42 mm apart and individual slice deformations introduce additional differences, it is only possible to roughly evaluate the same anatomical regions. The 3D-sSAXS inclinations were therefore not evaluated in the same four square regions as the angular ComSLI inclinations, but in larger areas of corpus callosum, fornix, and cingulum (bottom/top) containing these regions.

Another difference between angular ComSLI and 3D-sSAXS inclination maps is that 3D-sSAXS provides a fiber inclination estimate for all (white matter) pixels, while the inclination angles in the angular ComSLI measurement were only computed for image pixels with one or two prominent peaks (indicative of parallel fibers, see Section 2.7.2); image pixels with more prominent peaks appear black in Figure 5d which makes the inclination map look darker in some places than the one from 3D-sSAXS. Table 1 shows the percentage of pixels in corpus callosum, fornix, and cingulum that have a certain number of prominent peaks. The angular ComSLI measurement was performed with a polar illumination angle of θ≈44° for better comparison with previous studies [27,29]. However, this implies that inclination angles larger than 68° could not be resolved ($\alpha_{\max}=90{}^{\circ}-\theta /2$), as shown in Appendix D, Figure A4f. These pixels have only one prominent peak (1.6% of pixels in the cingulum, see Table 1), and are displayed yellow in (d), while they are displayed with different ranges of yellow in (e). Crossing regions with 4 or 6 prominent peaks as they are mostly found in the cingulum (23.5% of the pixels) were not evaluated as the current model assumes non-crossing fibers. 3, 5 or ≥7 prominent peaks are indicative of more complex structures (e.g., several crossing inclined fibers) or of noise in the scattering profile. While most pixels in the corpus callosum (92.6%) and fornix (99.5%) contain two prominent peaks, indicative of in-plane or moderately inclined fibers, the cingulum is known to contain highly inclined, but also multiple crossing fibers, which are difficult to reconstruct with the current model.

Despite these differences, the histograms in Figure 5f,g show similar tendencies regarding the fiber inclinations in the investigated regions: The histograms of corpus callosum and fornix show similar value distributions, while the bottom cingulum yields a broader distribution for the 3D-sSAXS measurement than for the angular ComSLI measurement. The top cingulum features high fiber inclinations in both measurements while the angular ComSLI measurement is clipped at 68° inclination. Comparing the results to the fitted inclinations obtained from the measured scattering patterns (colored vertical lines in the histograms), the fitted inclinations of corpus callosum and cingulum are compatible with the fiber inclinations in the histograms, while the inclinations in the fornix seem to be actually smaller than the fitted inclination obtained from the scattering pattern. However, it should be noted that the scattering patterns were measured up to a polar angle of 26°, while the angular ComSLI measurement was performed with a polar illumination angle of 44°, which naturally leads to differences. Another observation we can make when comparing the measured scattering patterns to the out-of-plane fiber orientations obtained from the 3D-sSAXS measurement is that the out-of-plane directions of the fibers determined by the 3D-sSAXS measurement (indicated by 3D-arrows in (e)) correspond to what we expect from the opening of the curvatures observed in the measured scattering patterns in (c): the opening of the curvature is in the direction in which the fibers lie closest to the LED-display, i.e., the image plane.

The results show that the fiber inclinations estimated from the measured scattering patterns for different brain regions seem plausible. Using angular ComSLI and estimating the fiber inclination from the azimuthal peak distance seems to give a first good guess of the out-of-plane fiber orientations, but is limited especially when it comes to larger fiber inclinations.

## 4. Discussion

Although previous simulations and experimental studies [35,36,40] suggest that inclined (nerve) fibers lead to a curved high-intensity line in ComSLI scattering patterns, a quantitative description with an analytical model has not been realized so far. Recent advances in two-photon polymerization (2PP) allowed us to 3D-nanoprint fiber bundles that mimic biological fibers (micrometer diameters, ≤2 µm inter-fiber gaps) with known underlying geometry. In this way, we were able to study how the scattering behavior depends on the fiber inclination while leaving all other parameters unchanged.

Compared to other light-based 3D fabrication approaches, such as digital-light processing [57] which is limited to several microns resolution, the sub-micrometric feature resolution of 2PP [46] is less affected by potential over-polymerization issues which would affect the quality of few microns diameters fibers and related inter-fiber gaps. 2PP recently enabled 3D-printing of nanopillar arrays with 500 nm diameter for neuromechanobiology studies [46]. Future advancements might include the use of biomaterials whose mechanical properties are closer to nerve fibers, such as photocrosslinkable hydrogels [58,59].

The 3D-printed fiber bundles used in this study are similar to those used in previous FDTD simulations [40]: the fibers are uniformly randomly distributed, have slightly different fiber diameters, and are naturally “grown”, i.e., not completely straight (cf. Appendix D, Figure A2). Although dimensions and growth patterns were chosen to mimic biological fibers as closely as possible, the 3D-printed phantoms differ from real biological fiber tissue and from the model used in the FDTD simulations [40]: First of all, to ensure stability during 3D-printing, the fibers were printed from a single solid material (IP-Dip2, n≈1.55) in air (n=1), leading to higher refractive index differences than in biological fiber tissues, where the fibers usually have a lower refractive index [60,61] and are surrounded by an aqueous solution or other materials with higher refractive indices than air. Measuring the phantoms in oil or water would have been technically challenging as fibers might detach or get damaged during liquid infiltration, and air bubbles could become trapped within the structure, leading to more artefacts. In addition, not all biological fibers can be modeled as solid fibers with a single refractive index: Nerve fibers (myelinated axons) consist of an inner axon core with lower refractive index than the outer myelin sheath. In the FDTD simulations, nerve fibers were modeled as inner axon (n=1.34), outer myelin sheaths (n=1.47), and surrounding glycerin/embedding solution (n=1.37). Second, the diameters of the printed fibers were (8±1.6) µm. Most nerve fibers in the investigated brain regions, like the corpus callosum, are expected to have diameters of 1 µm or less [42,43,44], while some nerve fibers can also have diameters of several microns. The FDTD simulations were therefore performed with 1−1.6 µm fiber diameters, which is closer to the illumination wavelength. Third, the 3D-printed phantoms have a different thickness (40 µm) than the measured monkey brain sections (60 µm); FDTD simulations were performed on cubes with 30 µm side length. Fourth, the FDTD simulations were performed with coherent light, while the ComSLI measurements were performed with mostly incoherent LED light. However, coherent Fourier scatterometry measurements already showed that the scattering patterns of a vervet brain section are expected to look similar to ComSLI scattering patterns when being measured with coherent (laser) light [62]. And previous studies also support an effectively incoherent description of the intensities detected in a ComSLI measurement [63].

Regarding the different refractive indices and fiber diameters, it can be stated that a larger refractive index contrast leads to a stronger averaged scattering intensity, while a larger fiber diameter leads to sharper peaks (smaller peak widths) [64]. However, neither of them is expected to change the position of the scattering peaks or the course of the high-intensity lines so that the conical diffraction model remains valid for different refractive index contrasts and fiber diameters. Apart from fiber diameters, other microstructural properties like packing density or orientation dispersion can also affect the width and prominence of the scattering peaks. For highly inclined fibers with small peak distances, regions with larger and more directed fibers exhibiting sharper and more prominent peaks might facilitate the determination of fiber inclinations.

Regarding the different thicknesses, previous studies [26,36] have shown that ComSLI applied to brain sections of different thicknesses (from 5 µm FFPE sections to 60 µm unstained cryo-sections) yields similar (normalized) scattering signals. However, it should be noted that these studies mostly focused on the reconstruction of in-plane fiber orientations, not out-of-plane orientations. Increasing the sample thickness generally increases the number of scattering structures encountered by the transmitted light and therefore the probability of higher-order scattering. While thicker samples can provide a stronger total scattering signal, they lead to increased attenuation and depth averaging. Thinner samples, on the other hand, can lead to a weaker scattering signal, but are expected to reduce multiple scattering and the superposition of different fiber populations along the optical axis. Thicker or thinner samples are therefore not necessarily better for determining the fiber inclination.

With increasing optical thickness, multiple scattering is expected to become increasingly relevant. Low-order scattering contributions are expected to retain more direct information about the local fiber geometry, whereas higher-order scattering can broaden the angular intensity distribution, reduce the contrast of distinct scattering maxima, and potentially affect the accuracy with which their positions, and therefore the fiber inclination, can be determined. However, multiple scattering in white matter is not necessarily isotropic: recent time- and space-resolved measurements by Pini et al. (2025) [65] demonstrated anisotropic diffuse light transport with a lower effective scattering rate and enhanced propagation along the local axonal orientation. This suggests that higher-order scattering may retain a relationship to the underlying fiber architecture, so that after a few scattering events the light will be transported along the nerve fibers and ComSLI might therefore only detect the light of “ballistic” or “few” scattering events.

Although the 3D-printed fibers can mimic biological fibers only to a certain extent, the measured scattering patterns of the 3D-printed phantoms and of the tilted corpus callosum showed a similar behavior for inclined fibers: While the nerve fiber bundles seem to scatter the light more broadly (Figure 4d vs. Figure 3d), the points of highest intensity (red dots) lie on the same line as predicted by the conical diffraction model for α={0,20,40,60}° (Figure 3f,g and Figure 4e,f). This shows that the 3D-printed phantoms can be used for modeling the scattering behavior on biological fibers. It should be noted that tilting the corpus callosum section is different from measuring a horizontal brain section with inclined fibers which are cut off at the bottom and top (as in the vervet brain section investigated in Figure 5). However, the comparison with the 3D-printed phantoms in which the fibers are also cut off shows that similar scattering patterns (middle high-intensity lines) are expected for the investigated inclinations.

The fiber inclinations estimated from the measured scattering patterns are very close to the theoretical expectation, both for the 3D-printed phantoms and the tilted corpus callosum. The differences between fitted and expected inclination values are 3° or less (Appendix C, Table A1 and Table A2); the differences between fitted and expected in-plane angles are 0.8° or less for the tilted corpus callosum (Appendix C, Table A3). We should note that the theoretical expectation for the tilted corpus callosum was based on an estimate (average) of fitted values, but we show that the model is self-consistent as the tilt angles of the measurements are known.

Our study shows that the highest-intensity points observed in the measured scattering patterns of inclined (nerve) fibers lie on conical diffraction curves up to 60° inclination (Figure 4). While we were able to show that the conical diffraction model still applies to higher inclination angles for 3D-printed fibers, we could not validate this behavior for biological fibers. For the validation, we tilted a mostly in-plane fiber bundle (corpus callosum in coronal vervet brain section) so that the maximum possible inclination angle was limited by the maximum tilt angle of the specimen stage. For tilt angles >60°, the specimen stage would block the incident light from the LED-display. The reason why we selected a mostly in-plane fiber bundle as starting point is that it allowed us to visually determine the in-plane fiber orientation and to properly align the fiber bundle with the *x*-axis to achieve the maximum possible fiber inclination.

Because of the limited tilt angle, we cannot confirm whether highly inclined nerve fibers (60°<α<90°) show a similar scattering behavior as the 3D-printed fibers, for which the high-intensity points of the scattering pattern lie on an out-of-center ellipse in agreement with the conical diffraction model, or whether they show a broader scattering pattern for α = 70° and 80° as predicted by the FDTD simulations [40]. For a vertical fiber bundle (α=90°), both the measured scattering patterns of the 3D-printed phantoms and the simulated scattering patterns of the FDTD simulations yield an isotropic scattering pattern with broad scattering around the center (cf. Figure 5b and [40] Figure 7a, respectively). And indeed, we observe a mostly isotropic scattering pattern for highly inclined nerve fibers as in the upper cingulum of the coronal vervet brain section (Figure 5c on the right), where the 3D-sSAXS measurement of a neighboring section 0.42 mm away yielded inclination angles of 80° and higher (Figure 5e). Again, it should be noted that it is only possible to roughly compare the same anatomical regions due to the distance and possible slice deformations.

We here present an automated way to estimate fiber inclinations from measured scattering patterns. However, not only is the measurement (acquiring 40×40 images) very time consuming, the data analysis also takes a lot of time due to the large amount of data. We therefore studied the potential of using angular ComSLI (requiring only 24 instead of 1600 images) to estimate the inclination from the azimuthal peak distance in the scattering profile of each image pixel, instead of analyzing full scattering patterns (see Figure 5d,f).

However, when estimating the inclination from the azimuthal peak distance, there is a theoretical limit given by the conical diffraction model (see Figure 1c and Appendix D, Figure A4f). The maximum inclination angle that can be determined is: $\alpha_{\max}=90{}^{\circ}-\theta /2$ (see Equation (A19) in Appendix B). This means that the larger the illumination angle θ, the smaller the maximum inclination that can be determined. For higher inclinations, diffraction occurs along an ellipse that is not intersected anymore by larger polar angles (see Figure 1b and Figure A4c). When looking at the relationship between azimuthal peak distance and inclination angle as predicted by the conical diffraction model (Figure 1c), we also see that the slope of this curve depends on the polar illumination angle. Ideally, the curve should be as steep as possible, so that the uncertainty in the peak distance, which is difficult to determine exactly, does not lead to large changes in the estimated inclination. However, this can only be obtained by a larger polar illumination angle, which leads to a lower $\alpha_{\max}$. The polar illumination angle of 44°, which is currently used for angular ComSLI measurements, is a good compromise (see dashed curve in Figure 1c): It allows a relatively good estimation of the inclination up to 60°. To better determine higher inclination angles, an additional measurement should be performed with a smaller polar illumination angle, e.g., θ=10° which allows to determine inclinations up to 85° (blue curve in Figure 1c). Note that there is also a practical limit regarding the minimum polar illumination angle, namely when the (unscattered) light directly shines into the camera.

To realize measurements with multiple polar angles, LED rings of different sizes could be used. To reduce measurement time, Hadamard illumination [63] could be applied to each angular measurement allowing to reduce exposure time by multiplexing multiple illumination directions at once. Another possibility to speed up the measurement could be to make use of compressed sensing; this might even allow to measure scattering patterns with lower resolution [66] but sufficient detail for conical diffraction curve fitting. However, even when measuring at multiple polar angles or full scattering patterns, it will still be difficult to accurately estimate the inclination for higher inclined fibers, as light is expected to scatter more broadly, i.e., not along a defined line, and peak locations become more difficult to determine. Determining the best illumination strategy and the best compromise between measurement time (number of images/illumination angles) and accuracy of estimated inclination angles will be the subject of future studies.

The measured scattering patterns shown in this study are the average of multiple image pixels (10 × 10 pixels or more, corresponding to an area of 30 × 30 µm^2^ or more). The averaging was done to study the overall behavior of inclined fibers and not to depend on local or statistical fluctuations. As the scattered light is mostly incoherent [63], the analytical model describing the behavior of the averaged scattering patterns is also valid assuming a smoothly varying geometry due to homogeneous tissue regions. The angular ComSLI measurement shown in Figure 5d shows the estimated inclination angles per image pixel and is thus more prone to noise. While the conical diffraction curves can be fitted to multiple highest-intensity points in a measured scattering pattern, the angular ComSLI measurement yields a 1D scattering signal per image pixel and the inclination is computed from the azimuthal peak distance in this line profile. As can be seen in Figure 5d, multiple image pixels could not be evaluated (shown in black) because they had more than two prominent peaks and thus no defined peak distance. This could either be caused by two or three crossing fiber bundles within a single pixel (leading to 4 or 6 prominent peaks) or noisy signals (leading to more than two prominent peaks). The current model can only be applied to a single bundle of parallel inclined fibers per pixel; extending the model to crossing inclined fibers will be the subject of further research.

Moreover, SLIX uses a simple peak-finding algorithm, optimized for in-plane fibers, in which discretization is accounted for by computing the geometric center of the peak tip [54]. It is not optimized for broader scattering peaks or inclined fibers. This means that the determined peak distances might not be completely accurate. More advanced algorithms, e.g., using smoothing and Gaussian fitting, could be employed to determine the peak distance more accurately, however, they will also increase the computing time and might not be feasible for larger fields of view.

Our study is a first proof of concept, showing that the high-intensity points of a measured scattering pattern for inclined biological fibers lie on the same curves as the diffraction peaks of an inclined grating. We here provide for the first time of our knowledge an analytical model (conical diffraction model) allowing to determine the fiber inclination from a measured scattering pattern, or possibly from a measured scattering signal. We show that the model accurately describes our measurement data up to 60° inclination. We used the model to determine theoretical limitations, like the cut-off angle for angular ComSLI, and to determine the best possible inclination estimate for real biological fibers under ideal conditions. We still need to evaluate our inclination estimation in practice, i.e., when performing a pixel-wise evaluation and when speeding up the measurement with angular ComSLI. Future studies need to be performed to investigate how accurate fiber inclinations can be estimated per image pixel in a reasonable amount of time, and how accurately we can determine the inclination angles of highly inclined fibers.

However, even if only a rough estimate of the fiber inclination angle is possible, it allows us to go from solely 2D-fiber orientations to 3D-orientations, and the estimate can be used, e.g., in fiber tractography algorithms to better follow the course of fibers across adjacent sections, and to better assess the (3D) collagen fiber orientation relative to tumor boundaries. It should be noted that this study only investigated regions with mostly parallel inclined fibers; regions with crossing inclined fibers are expected to show a more complex scattering behavior as the scattering patterns of different 3D-fiber orientations overlay (cf. Figure F3 in [34]). A reliable reconstruction of multiple 3D-fiber orientations per image pixel is therefore much more challenging and requires further research.

In this study, we have only validated our results on brain sections (nerve fibers). However, the nerve fibers showed a similar scattering behavior as the 3D-printed fibers, which have larger diameters and lack an inner core with different refractive index. This suggests that our findings can be generalized to muscle fibers, which also have no inner core and larger diameters, and possibly to other types of fibers like collagen.

## 5. Conclusions

ComSLI is a versatile technique for mapping biological fibers in histological tissue sections: it allows microscopic fiber mapping across large fields of view also in crossing regions, is easy to implement, fast when using angular ComSLI, and compatible with various sample preparations and staining. However, an accurate determination of fiber orientations was so far only possible for in-plane (2D) orientations. Here, we present for the first time of our knowledge an analytical (conical diffraction) model that can be used to determine the out-of-plane fiber inclinations from ComSLI measurements in regions with non-crossing fibers. Using 3D-nanoprinted fiber bundles with different inclination angles, we showed that the highest-intensity points of ComSLI scattering patterns lie along the same curved lines as the diffraction orders of inclined gratings, and that the underlying fiber inclinations can be reconstructed with an accuracy of 3° or higher by fitting conical diffraction curves to these highest-intensity points. We validated our results on biological samples (tilted nerve fiber bundle) up to 60° inclination, and showed that the conical diffraction model can be used to estimate inclinations of non-crossing fibers in a vervet monkey brain section. It is expected that our findings can be generalized to other types of fibers, like collagen or muscle fibers. While the fiber inclinations were determined with high accuracy from scattering patterns obtained with ComSLI scatterometry, we also demonstrated the potential of using the much faster angular ComSLI for estimating fiber inclinations from azimuthal peak distances in non-crossing fiber regions. In comparison to scatterometry, the determined fiber inclinations turned out to be more error-prone and there is a cut-off for high inclination angles. Further studies are needed to find the best compromise between the number of illumination angles and the measurement time, and to better quantify the behavior of highly inclined (α>60°) and crossing inclined fibers.

With the provided analytical model, we are finally able to extend ComSLI from 2D-fiber mapping to 3D-fiber mapping. This will open up other applications like facilitating fiber tractography across 3D-volumes (adjacent sections) or allowing a more accurate determination of collagen fiber angles relative to tumor boundaries.

## Figures and Tables

**Table 1:** Percentage of pixels in the inclination map (Figure 5d) that have a certain number of prominent peaks, evaluated for the entire image and for the indicated white matter regions: corpus callosum, fornix, cingulum (surrounded by cyan, green, and magenta lines in Figure 5a and Figure 5d, respectively).

Region	1 Peak	2 Peaks	3 Peaks	4 Peaks	5 Peaks	6 Peaks	≥7 Peaks
entire image	0.08%	45.69%	11.42%	13.92%	12.64%	9.72%	6.54%
corpus callosum	0.00%	92.64%	4.51%	2.57%	0.22%	0.05%	0.01%
fornix	0%	99.53%	0.41%	0.04%	0.01%	0.00%	0.00%
cingulum	1.65%	34.67%	27.49%	18.50%	10.31%	5.00%	2.38%

**Figure A1:**
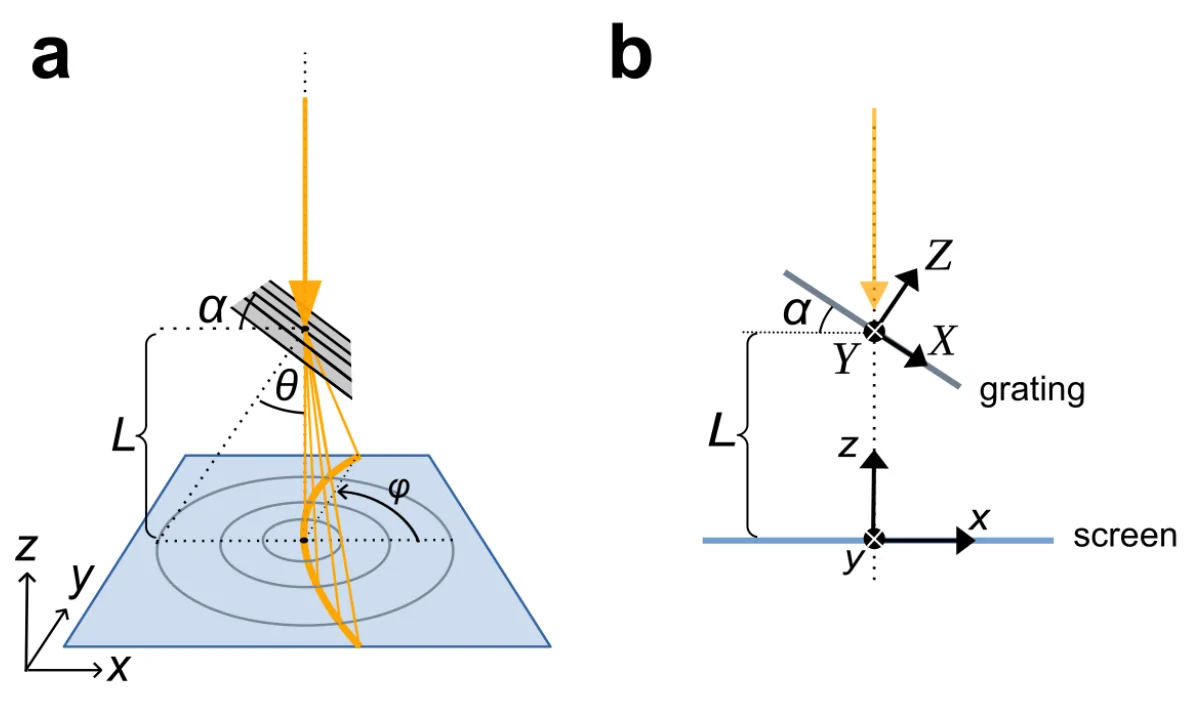
Definition of coordinate systems. (**a**) Schematic showing the diffraction at a grating with inclination angle α (main Figure 1a). (**b**) Local coordinate system (X,Y,Z) of the grating and coordinate system (x,y,z) of the screen. The *X*-axis points along the grating slits, the *Y*-axis perpendicular to the grating slits (in the same direction as the *y*-axis), and the *Z*-axis along the grating normal. The coordinate systems are rotated by an angle α with respect to each other, and their origins lie at a distance *L* apart. The orange arrows indicate the direction of the incident light.

## Data Availability

The original data presented in the study are openly available in Zenodo at https://doi.org/10.5281/zenodo.22643344.

## Abbreviations

The following abbreviations are used in this manuscript:

CMOS	complementary metal-oxide-semiconductor
ComSLI	computational scattered light imaging
dMRI	diffusion magnetic resonance imaging
FDTD	finite-difference time-domain
FFPE	formalin-fixed paraffin-embedded
LED	light emitting diode
pSHG	polarimetric second harmonic generation
(PS-)OCT	(polarization-sensitive) optical coherence tomography
RGB	red, green, blue
RMSD	root-mean-square distance
SLIX	scattered light imaging toolbox
2D/3D	two-/three-dimensional
2PP	two-photon polymerization
3D-PLI	three-dimensional polarized light imaging
3D-sSAXS	3D-scanning small-angle X-ray scattering

## Appendix A. Use of the Conical Diffraction Model

This study shows that the highest scattering intensities of inclined (nerve) fiber bundles lie on the same conical diffraction curves as the diffraction orders of inclined gratings, although biological fibers are randomly spaced and not perfectly parallel unlike a grating. An explanation for this phenomenon is that conical diffraction occurs already for single inclined fibers, without any spatial periodicity of the scatterer. This was also confirmed in previous Rayleigh-Gans calculations on single inclined cylinders [63], where the high-intensity lines in the simulated scattering patterns followed the same conical diffraction curves.

For a cylindrical fiber with axis $\vec{u}$ that is sufficiently long to be approximately translationally invariant along the fiber axis, the wave-vector component parallel to the fiber is approximately conserved: $\left(\vec{k_{s}}-\vec{k_{i}}\right)\cdot \vec{u}\approx 0$, with $\vec{k_{i}}$ and $\vec{k_{s}}$ being the incident and scattered wave vectors, respectively. Assuming elastic scattering $\left(|\vec{k_{i}}|=|\vec{k_{s}}|\right)$, this implies that the directions of scattered light $\vec{k_{s}}$ are restricted to a cone around the fiber axis. A diffraction grating with grooves parallel to $\vec{u}$ obeys the same constraint, but its periodicity perpendicular to the grooves leads to discrete diffraction orders along this cone. A biological fiber bundle, which contains randomly spaced fibers with finite lengths and slightly different orientations, where the individual scattering distributions share essentially the same directional dependence, will rather produce a continuous, broadened intensity distribution than discrete orders. The limited coherence of the LED illumination used in ComSLI additionally reduces interference between contributions from different fibers and promotes an effectively incoherent intensity superposition.

## Appendix B. Derivation of Formulas

In the following, we derive the formulas used to describe the diffraction at an inclined 2D-grating (main Figure 1b) and the corresponding azimuthal peak distances (main Figure 1c).

For the derivation, we assume the setting shown in main Figure 1a (or Figure A1a): The coordinate system (x,y,z) is defined such that the *x*- and *y*-axes span the detector screen, and the *z*-axis points from the screen towards the grating. The origin lies in the center of the screen. The grating is oriented such that the *y*-axis lies perpendicular to the slits, and the *x*-axis parallel to the projection of the slits onto the screen. The grating is rotated (inclined) around the *y*-axis by an angle α following the right-hand rule. Light falls onto the grating from above, along the *z*-axis. A point on the screen can be described by (x,y) as well as by spherical coordinates θ (polar angle) and φ (azimuthal angle) defined relative to the center of the grating (where the light hits the grating). We here only consider the case in which the grating slits (or their projection) are oriented along the *x*-axis. If the grating is rotated around the *z*-axis by an angle φ, the diffraction pattern is rotated accordingly.

In addition to the coordinate system of the screen (x,y,z), we define the local coordinate system of the grating (X,Y,Z) (see Figure A1b). In this coordinate system, the *X*-axis points in the direction of the grating slits, the *Y*-axis perpendicular to the slits (i.e., in the same direction as the *y*-axis), and the *Z*-axis along the grating normal. The origin of this coordinate system lies in the center of the grating, at a distance z=L above the screen.

### Appendix B.1. Conical Diffraction at an Inclined Grating

Harvey & Pfisterer (2019) [49] provide a grating equation that describes diffraction on a grating in the case that the light strikes the grating at a large oblique angle (conical diffraction). Using their Equations (4) and (5) and writing:

$$\begin{array}{cc} \phi_{0}=\alpha & (\mathrm{angle}\;\mathrm{between}\;\mathrm{grating}\;\mathrm{normal}\;\mathrm{and}\;\mathrm{incident}\;\mathrm{light}),\\ \theta_{0}=0 & (\mathrm{angle}\;\mathrm{between}\;\mathrm{grating}\;\mathrm{normal}\;\mathrm{and}\;\mathrm{screen}\;\mathrm{normal}),\\ \theta_{m}=\theta & (\mathrm{not}\;\mathrm{considering}\;\mathrm{discrete}\;\mathrm{diffraction}\;\mathrm{orders}),\\ \beta_{m}=X/(\sqrt{X^{2}+Y^{2}+Z^{2}}) & (\mathrm{definition}\;\mathrm{of}\;\mathrm{direction}\;\mathrm{cosine}\;\mathrm{along}\;\mathrm{the}\;\mathrm{grating}\;\mathrm{slits}\\ & \mathrm{in}\;\mathrm{the}\;\mathrm{local}\;\mathrm{coordinate}\;\mathrm{system}\;\mathrm{of}\;\mathrm{the}\;\mathrm{grating}), \end{array}$$

we obtain the following equation for $\beta_{m}$:

(A1) $$\begin{array}{cc} \frac{X}{\sqrt{X^{2}+Y^{2}+Z^{2}}} & =\sin(\alpha ) \end{array}$$

(A2) $$\Leftrightarrow X^{2}\cos^{2}(\alpha )=\left(Y^{2}+Z^{2}\right)\sin^{2}(\alpha ),$$

using $\left(1-\sin^{2}\alpha \right)=\cos^{2}\alpha$. This equation describes a cone in the local coordinate system of the grating (X,Y,Z), i.e, the cone axis is aligned with the slits of the grating (the *X*-direction).

### Appendix B.2. Intersection with the Detector Plane

In order to describe the conical diffraction curves observed on the screen, we need to transform the coordinates to get from the local coordinate system of the grating (X,Y,Z) to the coordinate system of the screen (x,y,z). The grating coordinate system is related to the screen coordinate system by rotating it around the *y*-axis by an angle α and moving the origin up to z=L; the *y*-axis remains unchanged:

(A3) X=xcos(α)+Lsin(α),

(A4) Y=y,

(A5) Z=−xsin(α)+Lcos(α).

Inserting this into Equation (A2) and using the following trigonometric identities:

(A6) 2sin(α)cos(α)=sin(2α),

(A7) $$\sin^{2}\left(\alpha \right)+\cos^{2}\left(\alpha \right)=1,$$

(A8) $$\cos^{4}\left(\alpha \right)-\sin^{4}\left(\alpha \right)=\cos^{2}\left(\alpha \right)-\sin^{2}\left(\alpha \right)=\cos\left(2\alpha \right),$$

we obtain the following equation, which describes the conical diffraction curves observed on the screen at distance *L* from the grating (see Figure 1b):

(A9) $$y^{2}\sin^{2}\left(\alpha \right)-xL\sin\left(2\alpha \right)-x^{2}\cos\left(2\alpha \right)=0$$

(A10) $$\Leftrightarrow \begin{array}{c} y=\pm \frac{\sqrt{xL\sin\left(2\alpha \right)+x^{2}\cos\left(2\alpha \right)}}{\sin\alpha } \end{array},\;\mathrm{for}\;0<|\alpha |<90{}^{\circ}.$$

Note that in the ComSLI setup, the distance *L* corresponds to the distance between the LED display and the sample.

The behavior for α=0° and α=90° follows from taking the limit of Equation (A9). Taking the α→0° limit, we obtain:

$$-x^{2}=0\Rightarrow x=0.$$

The diffraction orders lie on a straight (vertical) line as expected for a non-inclined grating. For the α→±90° limit, we obtain:

$$y^{2}+x^{2}=0\Rightarrow \left(x,y\right)=\left(0,0\right).$$

The light is not diffracted and we observe a single bright dot at the origin of the screen.

### Appendix B.3. Azimuthal Peak Distance

Relative to the origin/center of the grating (where the light hits the grating), a point (x,y) on the detector screen can be described by the polar angle θ and the in-plane azimuthal angle φ (cf. main Figure 1a):

(A11) x=Ltan(θ)cos(φ),y=Ltan(θ)sin(φ).

With this, the above Equation (A9) can be written in terms of spherical coordinates. After dividing by $L^{2}$ and by tanθ (considering only cases in which 0<θ<90°), we obtain:

(A12) $$\cos^{2}\left(\varphi \right)\tan\left(\theta \right)\cos^{2}\left(\alpha \right)+\cos\left(\varphi \right)\sin\left(2\alpha \right)-\tan\left(\theta \right)\sin^{2}\left(\alpha \right)=0,$$

where we used: $\sin^{2}\left(\varphi \right)=1-\cos^{2}\left(\varphi \right)$, and $\cos\left(2\alpha \right)=\cos^{2}\left(\alpha \right)-\sin^{2}\left(\alpha \right)$.

This is a quadratic equation in the form $ax^{2}+bx+c=0$. The solution is given by: $x=\frac{-b\pm \sqrt{b^{2}-4ac}}{2a}$. Hence, we obtain (for x=cosφ and assuming 0<|α|<90°):

(A13) $$\begin{array}{c} \cos\left(\varphi \right)=\frac{-\sin\left(2\alpha \right)\pm \sqrt{\sin^{2}\left(2\alpha \right)+4\tan^{2}\left(\theta \right)\cos^{2}\left(\alpha \right)\sin^{2}\left(\alpha \right)}}{2\tan\left(\theta \right)\cos^{2}\left(\alpha \right)}. \end{array}$$

Writing sin(2α)=2sin(α)cos(α) and $\frac{\sin(\alpha )}{\cos(\alpha )}=\tan\left(\alpha \right)$, we can further simplify:

(A14) $$\begin{array}{c} \cos\left(\varphi \right)=\tan\left(\alpha \right)\frac{\pm \sqrt{1+\tan^{2}\left(\theta \right)}-1}{\tan(\theta )}. \end{array}$$

Solving this for *α* yields:

(A15) $$\begin{array}{c} \tan\left(\alpha \right)=\frac{\tan(\theta )\cos(\varphi )}{\pm \sqrt{1+\tan^{2}\left(\theta \right)}-1}. \end{array}$$

Considering the situation depicted in main Figure 1b, where the grating slits (or their projection) are aligned with the *x*-axis, we only need to consider the upper part of the curves and the azimuthal peak distance is given by: Δφ=2|φ|.

Inserting this into the above equation, we see that the azimuthal peak distance Δφ at a given polar angle 0<θ<90° can be used to estimate the (absolute) inclination angle |α| of the grating (see Figure 1c):

(A16) $$\begin{array}{c} \left|\alpha \right|=\arctan\left(\frac{\tan\left(\theta \right)\cdot \cos\left(\frac{\Delta \varphi }{2}\right)}{\sqrt{1+\tan^{2}\left(\theta \right)}-1}\right) \end{array}.$$

From this equation, the maximum resolvable inclination angle $\alpha_{\max}$ can be obtained by considering the limiting case Δφ=0, in which the two scattering peaks merge. In this case, cos(Δφ/2)=1, yielding:

(A17) $$\alpha_{\max}=\arctan\left(\frac{\tan\theta }{\sqrt{1+\tan^{2}\theta }-1}\right)=\arctan\left(\frac{\sin\theta }{1-\cos\theta }\right),$$

using the trigonometric identity $\sqrt{1+\tan^{2}\theta }=\frac{1}{\cos\theta }$. Applying the half-angle identities sinθ=2sin(θ/2)cos(θ/2) and $1-\cos\theta =2\sin^{2}\left(\theta /2\right)$, this becomes:

(A18) $$\alpha_{\max}=\arctan\left(\frac{2\sin\left(\frac{\theta }{2}\right)\cos\left(\frac{\theta }{2}\right)}{2\sin^{2}\left(\frac{\theta }{2}\right)}\right)=\arctan\left(\cot\left(\frac{\theta }{2}\right)\right).$$

Finally, using cot(x)=tan(90°−x) and noting that 0<θ/2<45°, we obtain the closed-form expression:

(A19) $$\begin{array}{c} \alpha_{\max}=90{}^{\circ}-\frac{\theta }{2} \end{array}.$$

## Appendix C. Fit Values

The tables below list the fit values obtained from fitting the conical diffraction curves to the measured scattering patterns in main Figure 3, Figure 4 and Figure 5:

$\varphi /\alpha_{\mathrm{fit}}$	fitted in-plane/inclination angles
Min/Max $\varphi /\alpha_{\mathrm{fit}}$	ranges for which the root-mean-square (RMS) distance differs <1% from the minimum (a measure of the precision of our fit)
Min RMSD	minimum RMS distance
Stddev RMSD	standard deviation of the RMS distance values
$\varphi /\alpha_{\mathrm{theory}}$	theoretical in-plane/inclination angles of the tilted vervet-cc sample, computed under the assumption that the zero-tilt fiber orientation corresponds to the average of the fitted in-plane/inclination angles back-rotated to their orientation before the tilt
$\varphi /\alpha_{\mathrm{backrotated}}$	fitted in-plane/inclination angles of the tilted vervet-cc sample, back-rotated to their orientation before the tilt

**Table A1:** Fit values for the 3D-printed phantoms for different fiber inclinations α (main Figure 3).

α (°)	$\alpha_{\mathrm{fit}}$ (°)	Min $\alpha_{\mathrm{fit}}$ (°)	Max $\alpha_{\mathrm{fit}}$ (°)	Min RMSD	Stddev RMSD
0	3.1	−2.7	8.7	0.35	0.17
20	18.5	12.8	23.9	0.42	0.28
40	41.0	37.8	44.0	0.31	0.16
60	61.1	59.4	62.7	0.66	0.32
80	82.6	82.3	82.9	0.69	0.43

**Table A2:** Fit values for the tilted vervet-cc inclinations for different tilt angles $\alpha^{\prime }$ (main Figure 4).

$\alpha^{\prime }$ (°)	$\alpha_{\mathrm{theory}}$ (°)	$\alpha_{\mathrm{fit}}$ (°)	Min $\alpha_{\mathrm{fit}}$ (°)	Max $\alpha_{\mathrm{fit}}$ (°)	Min RMSD	Stddev RMSD
0	−4.29	−6.3	−14.2	0.9	0.15	0.08
20	15.68	14.0	6.9	20.6	0.11	0.06
40	35.65	38.6	34.6	42.1	0.12	0.07
60	55.59	56.3	54.2	58.2	0.14	0.09

**Table A3:** Fit values for the tilted vervet-cc in-plane orientations and the back-rotated values for different tilt angles $\alpha^{\prime }$ (main Figure 4).

$\alpha^{\prime }$ (°)	$\varphi_{\mathrm{theory}}$ (°)	$\varphi_{\mathrm{fit}}$ (°)	Min $\varphi_{\mathrm{fit}}$ (°)	Max $\varphi_{\mathrm{fit}}$ (°)	$\varphi_{\mathrm{backrotated}}$ (°)	$\alpha_{\mathrm{backrotated}}$ (°)
0	2.98	2.8	1.8	3.8	2.80	−6.30
20	3.09	2.6	1.7	3.4	2.54	−5.98
40	3.66	4.1	3.1	5.0	3.20	−1.33
60	5.26	6.1	5.1	7.1	3.39	−3.54

**Table A4:** Fit values for the vervet region inclinations (main Figure 5c).

Region	$\alpha_{\mathrm{fit}}$ (°)	Min $\alpha_{\mathrm{fit}}$ (°)	Max $\alpha_{\mathrm{fit}}$ (°)	Min RMSD	Stddev RMSD
corpus callosum	−3.1	−9.2	3.1	0.16	0.12
fornix	−31.6	−35.8	−28.6	0.16	0.09
cingulum (bottom)	33.9	27.1	39.8	0.44	0.25

**Table A5:** Fit values for the vervet region in-plane orientations (main Figure 5c).

Region	$\varphi_{\mathrm{fit}}$ (°)	Min $\varphi_{\mathrm{fit}}$ (°)	Max $\varphi_{\mathrm{fit}}$ (°)
corpus callosum	2.6	1.4	3.9
fornix	−68.8	−69.8	−67.8
cingulum (bottom)	−73.2	−74.8	−71.7

## References

[1] Frontera W.R., Ochala J. (2015). Skeletal Muscle: A Brief Review of Structure and Function. Calcif. Tissue Int..

[2] Kesson M., Atkins E. (2005). Collagen fibres. Orthopaedic Medicine.

[3] Conklin M.W., Eickhoff J.C., Riching K.M., Pehlke C.A., Eliceiri K.W., Provenzano P.P., Friedl A., Keely P.J. (2011). Aligned Collagen Is a Prognostic Signature for Survival in Human Breast Carcinoma. Am. J. Pathol..

[4] Amunts K., DeFelipe J., Pennartz C., Destexhe A., Migliore M., Ryvlin P., Furber S., Knoll A., Bitsch L., Bjaalie J.G. (2022). Linking Brain Structure, Activity, and Cognitive Function through Computation. eNeuro.

[5] Cocks E., Taggart M., Rind F., White K. (2018). A guide to analysis and reconstruction of serial block face scanning electron microscopy data. J. Microsc..

[6] Peddie C.J., Collinson L.M. (2014). Exploring the third dimension: Volume electron microscopy comes of age. Micron.

[7] Eberle A.L., Zeidler D. (2018). Multi-Beam Scanning Electron Microscopy for High-Throughput Imaging in Connectomics Research. Front. Neuroanat..

[8] Amunts K., Lepage C., Borgeat L., Mohlberg H., Dickscheid T., Rousseau M.É., Bludau S., Bazin P.L., Lewis L.B., Oros-Peusquens A.M. (2013). BigBrain: An Ultrahigh-Resolution 3D Human Brain Model. Science.

[9] Carriel V., Campos A., Alaminos M., Raimondo S., Geuna S., Pellicciari C., Biggiogera M. (2017). Staining Methods for Normal and Regenerative Myelin in the Nervous System. Histochemistry of Single Molecules: Methods and Protocols.

[10] Schurr R., Mezer A.A. (2021). The Glial Framework Reveals White Matter Fiber Architecture in Human and Primate Brains. Science.

[11] Khan A.R., Cornea A., Leigland L.A., Kohama S.G., Jespersen S.N., Kroenke C.D. (2015). 3D Structure Tensor Analysis of Light Microscopy Data for Validating Diffusion MRI. NeuroImage.

[12] Sorelli M., Di Meo D., Bradley S., Cheli F., Ramazzotti J., Caria F.F., Perego L., Destrieux C., Hof P.R., Pavone F.S. (2025). Myelinated fiber labeling and orientation mapping of the human brain with light-sheet fluorescence microscopy. NeuroImage.

[13] Costantini I., Baria E., Sorelli M., Matuschke F., Giardini F., Menzel M., Mazzamuto G., Silvestri L., Cicchi R., Amunts K. (2021). Autofluorescence Enhancement for Label-Free Imaging of Myelinated Fibers in Mammalian Brains. Sci. Rep..

[14] Wang H., Lenglet C., Akkin T. (2015). Structure Tensor Analysis of Serial Optical Coherence Scanner Images for Mapping Fiber Orientations and Tractography in the Brain. J. Biomed. Opt..

[15] Dell’Acqua F., Tournier J.D. (2019). Modelling white matter with spherical deconvolution: How and why?. NMR Biomed..

[16] Jeurissen B., Descoteaux M., Mori S., Leemans A. (2019). Diffusion MRI fiber tractography of the brain. NMR Biomed..

[17] Damon B.M., Froeling M., Buck A.K.W., Oudeman J., Ding Z., Nederveen A.J., Bush E.C., Strijkers G.J. (2017). Skeletal muscle diffusion tensor-MRI fiber tracking: Rationale, data acquisition and analysis methods, applications and future directions. NMR Biomed..

[18] Raya J.G., Duarte A., Wang N., Mazzoli V., Jaramillo D., Blamire A.M., Dietrich O. (2024). Applications of Diffusion-Weighted MRI to the Musculoskeletal System. J. Magn. Reson. Imaging.

[19] Wang H., Magnain C., Wang R., Dubb J., Varjabedian A., Tirrell L.S., Stevens A., Augustinack J.C., Konukoglu E., Aganj I. (2018). as-PSOCT: Volumetric microscopic imaging of human brain architecture and connectivity. NeuroImage.

[20] Jones R., Grisot G., Augustinack J., Magnain C., Boas D.A., Fischl B., Wang H., Yendiki A. (2020). Insight into the fundamental trade-offs of diffusion MRI from polarization-sensitive optical coherence tomography in ex vivo human brain. NeuroImage.

[21] Axer M., Grässel D., Kleiner M., Dammers J., Dickscheid T., Reckfort J., Hütz T., Eiben B., Pietrzyk U., Zilles K. (2011). High-Resolution Fiber Tract Reconstruction in the Human Brain by Means of Three-Dimensional Polarized Light Imaging. Front. Neuroinform..

[22] Schmitz D., Amunts K., Lippert T., Axer M. A Least Squares Approach for the Reconstruction of Nerve Fiber Orientations from Tiltable Specimen Experiments in 3D-PLI. Proceedings of the 2018 IEEE 15th International Symposium on Biomedical Imaging (ISBI 2018).

[23] Axer M., Strohmer S., Grässel D., Bücker O., Dohmen M., Reckfort J., Zilles K., Amunts K. (2016). Estimating Fiber Orientation Distribution Functions in 3D-Polarized Light Imaging. Front. Neuroanat..

[24] Takemura H., Palomero-Gallagher N., Axer M., Grässel D., Jorgensen M.J.J., Woods R., Zilles K. (2020). Anatomy of nerve fiber bundles at micrometer-resolution in the vervet monkey visual system. eLife.

[25] Blanke N., Chang S., Novoseltseva A., Wang H., Boas D.A., Bigio I.J. (2023). Multiscale label-free imaging of myelin in human brain tissue with polarization-sensitive optical coherence tomography and birefringence microscopy. Biomed. Opt. Express.

[26] Georgiadis M., auf der Heiden F., Abbasi H., Ettema L., Nirschl J., Taghavi H.M., Wakatsuki M., Liu A., Ho W.H.D., Carlson M. (2025). Micron-resolution fiber mapping in histology independent of sample preparation. Nat. Commun..

[27] Abbasi H., Ettema L., van Elk R., Eskes M., Doukas M., Koppes S.A., Keereweer S., Menzel M. (2026). Whole-slide mapping of tumor tissue fiber architecture via computational scattered light imaging. Biomed. Opt. Express.

[28] Blanke N. (2023). Birefringence Microscopy for High-Resolution Imaging of Myelinated Axons and Myelin Pathology in the Postmortem Brain. Ph.D. Thesis.

[29] Ettema L., Mažeika V., Alizadeh M., Abbasi H., Barzda V., Menzel M. (2026). Fibre orientations in collagen-containing tissues revealed with computational scattered light imaging and polarimetric second harmonic generation microscopy. Sci. Rep..

[30] Georgiadis M., Schroeter A., Gao Z., Guizar-Sicairos M., Liebi M., Leuze C., McNab J.A., Balolia A., Veraart J., Ades-Aron B. (2021). Nanostructure-Specific X-Ray Tomography Reveals Myelin Levels, Integrity and Axon Orientations in Mouse and Human Nervous Tissue. Nat. Commun..

[31] Silva H., Tassone C., Ross E.G., Lee J.T., Zhou W., Nelson D. (2022). Collagen Fibril Orientation in Tissue Specimens From Atherosclerotic Plaque Explored Using Small Angle X-Ray Scattering. J. Biomech. Eng..

[32] Liebi M., Georgiadis M., Menzel A., Schneider P., Kohlbrecher J., Bunk O., Guizar-Sicairos M. (2015). Nanostructure Surveys of Macroscopic Specimens by Small-Angle Scattering Tensor Tomography. Nature.

[33] Georgiadis M., Schroeter A., Gao Z., Guizar-Sicairos M., Novikov D.S., Fieremans E., Rudin M. (2020). Retrieving neuronal orientations using 3D scanning SAXS and comparison with diffusion MRI. NeuroImage.

[34] Menzel M., Reuter J., Gräßel D., Huwer M., Schlömer P., Amunts K., Axer M. (2021). Scattered Light Imaging: Resolving the substructure of nerve fiber crossings in whole brain sections with micrometer resolution. NeuroImage.

[35] Menzel M., Ritzkowski M., Reuter J.A., Gräßel D., Amunts K., Axer M. (2021). Scatterometry Measurements With Scattered Light Imaging Enable New Insights Into the Nerve Fiber Architecture of the Brain. Front. Neuroanat..

[36] Menzel M., Gräßel D., Rajkovic I., Zeineh M.M., Georgiadis M. (2023). Using light and X-ray scattering to untangle complex neuronal orientations and validate diffusion MRI. eLife.

[37] Sporns O. (2013). The Human Connectome: Origins and Challenges. NeuroImage.

[38] Glasser M.F., Smith S.M., Marcus D.S., Andersson J.L.R., Auerbach E.J., Behrens T.E.J., Coalson T.S., Harms M.P., Jenkinson M., Moeller S. (2016). The Human Connectome Project’s Neuroimaging Approach. Nat. Neurosci..

[39] Ouellette J.N., Drifka C.R., Pointer K.B., Liu Y., Lieberthal T.J., Kao W.J., Kuo J.S., Loeffler A.G., Eliceiri K.W. (2021). Navigating the Collagen Jungle: The Biomedical Potential of Fiber Organization in Cancer. Bioengineering.

[40] Menzel M., Axer M., De Raedt H., Costantini I., Silvestri L., Pavone F.S., Amunts K., Michielsen K. (2020). Toward a High-Resolution Reconstruction of 3D Nerve Fiber Architectures and Crossings in the Brain Using Light Scattering Measurements and Finite-Difference Time-Domain Simulations. Phys. Rev. X.

[41] Nix J.S., Moore S.A. (2020). What Every Neuropathologist Needs to Know: The Muscle Biopsy. J. Neuropathol. Exp. Neurol..

[42] Aboitiz F., Scheibel A.B., Fisher R.S., Zaidel E. (1992). Fiber composition of the human corpus callosum. Brain Res..

[43] Aboitiz F., Ide A., Olivares R., Zaidel E., Iacoboni M. (2003). Corpus callosum morphology in relation to cerebral asymmetries in the postmortem human. The Parallel Brain: The Cognitive Neuroscience of the Corpus Callosum.

[44] Liewald D., Miller R., Logothetis N., Wagner H.J., Schüz A. (2014). Distribution of axon diameters in cortical white matter: An electron-microscopic study on three human brains and a macaque. Biol. Cybern..

[45] Puetzer J.L., Ma T., Sallent I., Gelmi A., Stevens M.M. (2021). Driving Hierarchical Collagen Fiber Formation for Functional Tendon, Ligament, and Meniscus Replacement. Biomaterials.

[46] Flamourakis G., Dong Q., Kromm D., Teurlings S., van Haren J., Allertz T., Smeenk H., de Vrij F.M.S., Tas R.P., Smith C.S. (2025). Deciphering the Influence of Effective Shear Modulus on Neuronal Network Directionality and Growth Cones’ Morphology via Laser-Assisted 3D-Printed Nanostructured Arrays. Adv. Funct. Mater..

[47] Sharaf A., Roos B., Timmerman R., Kremers G.J., Bajramovic J.J., Accardo A. (2022). Two-Photon Polymerization of 2.5D and 3D Microstructures Fostering a Ramified Resting Phenotype in Primary Microglia. Front. Bioeng. Biotechnol..

[48] Harvey J., Vernold C. (1998). Description of Diffraction Grating Behavior in Direction Cosine Space. Appl. Opt..

[49] Harvey J., Pfisterer R. (2019). Understanding diffraction grating behavior: Including conical diffraction and Rayleigh anomalies from transmission gratings. Opt. Eng..

[50] Vankevych P.P., Drobenko B.D., Ftomyn N.Y., Chornodolskyy Y.M., Dehtiarenko V.V., Sliusarenko A.V., Chernenko A.D., Bolkot P.A. (2022). Determining the position of a radiation source using the conical diffraction method. J. Phys. Stud..

[51] Matuschke F., Amunts K., Axer M. (2021). fastPLI: A Fiber Architecture Simulation Toolbox for 3D-PLI. J. Open Source Softw..

[52] Menzel M., Grässel D., Rajkovic I., Zeineh M., Georgiadis M. (2022). Dataset: Using light and X-ray scattering to untangle complex neuronal orientations and validate diffusion MRI. Zenodo.

[53] Virtanen P., Gommers R., Oliphant T.E., Haberland M., Reddy T., Cournapeau D., Burovski E., Peterson P., Weckesser W., Bright J. (2020). SciPy 1.0: Fundamental Algorithms for Scientific Computing in Python. Nat. Methods.

[54] Reuter J.A., Menzel M. (2020). SLIX: A Python package for fully automated evaluation of Scattered Light Imaging measurements on brain tissue. J. Open Source Softw..

[55] Shah A., Jhawar S., Goel A., Goel A. (2021). Corpus Callosum and Its Connections: A Fiber Dissection Study. World Neurosurg..

[56] Decramer T., Swinnen S., van Loon J., Janssen P., Theys T. (2018). White matter tract anatomy in the rhesus monkey: A fiber dissection study. Brain Struct. Funct..

[57] Sheng L., Li M., Zheng S., Qi J. (2022). Adjusting the accuracy of PEGDA-GelMA vascular network by dark pigments via digital light processing printing. J. Biomater. Appl..

[58] Chang S.Y., Ching T., Hashimoto M. (2022). Bioprinting using PEGDMA-based hydrogel on DLP printer. Mater. Today Proc..

[59] van Altena P.F.J., Ransanz L.C., Manco M., Heine V.M., Accardo A. (2025). Micro-digital light processing of conventional and hollow Gyroid mesoscale hydrogel scaffolds for neural cell cultures. Micro Nano Eng..

[60] Martins I.S., Silva H.F., Lazareva E.N., Chernomyrdin N.V., Zaytsev K.I., Oliveira L.M., Tuchin V.V. (2023). Measurement of tissue optical properties in a wide spectral range: A review [Invited]. Biomed. Opt. Express.

[61] de Campos Vidal B., Mello M.L.S., Caseiro-Filho A.C., Godo C. (1980). Anisotropic properties of the myelin sheath. Acta Histochem..

[62] Menzel M., Pereira S.F. (2020). Coherent Fourier scatterometry reveals nerve fiber crossings in the brain. Biomed. Opt. Express.

[63] Scheidt D., Matuschke F., Amunts K., Menzel M., Axer M. (2026). Hadamard-encoded scattered light imaging for faster, signal-enhanced mapping of brain fiber orientations. Biomed. Opt. Express.

[64] Scheidt D., Voß C., Rosero Arias C., Saavedra Castano S., Matuschke F., Koojimans R., Amunts K., Susarrey-Arce A., Axer M. (2026). 3D scattered light imaging: Extracting 3D fiber orientations from 1D line profiles in brain imaging. arXiv.

[65] Pini E., Meo D.D., Costantini I., Sorelli M., Bradley S., Wiersma D.S., Pavone F.S., Pattelli L. (2025). Anisotropic light propagation in human brain white matter. Neurophotonics.

[66] auf der Heiden F., Münzer O., van Staalduine S., Amunts K., Axer M., Menzel M., Tsia K.K., Goda K. (2024). Towards high-speed computational scattered light imaging by introducing compressed sensing for optimized illumination. Proceedings of the High-Speed Biomedical Imaging and Spectroscopy IX.

